# Morphogenesis of the *C*. *elegans* Intestine Involves Axon Guidance Genes

**DOI:** 10.1371/journal.pgen.1005950

**Published:** 2016-04-01

**Authors:** Alparsan Asan, Stephan A. Raiders, James R. Priess

**Affiliations:** 1 Fred Hutchinson Cancer Research Center, Seattle, Washington, United States of America; 2 Molecular and Cellular Biology Program, University of Washington, Seattle, Washington, United States of America; 3 Department of Biology, University of Washington, Seattle, Washington, United States of America; Harvard University, UNITED STATES

## Abstract

Genetic and molecular studies have provided considerable insight into how various tissue progenitors are specified in early embryogenesis, but much less is known about how those progenitors create three-dimensional tissues and organs. The *C*. *elegans* intestine provides a simple system for studying how a single progenitor, the E blastomere, builds an epithelial tube of 20 cells. As the E descendants divide, they form a primordium that transitions between different shapes over time. We used cell contours, traced from confocal optical z-stacks, to build a 3D graphic reconstruction of intestine development. The reconstruction revealed several new aspects of morphogenesis that extend and clarify previous observations. The first 8 E descendants form a plane of four right cells and four left cells; the plane arises through oriented cell divisions and VANG-1/Van Gogh-dependent repositioning of any non-planar cells. LIN-12/Notch signaling affects the left cells in the E8 primordium, and initiates later asymmetry in cell packing. The next few stages involve cell repositioning and intercalation events that shuttle cells to their final positions, like shifting blocks in a Rubik’s cube. Repositioning involves breaking and replacing specific adhesive contacts, and some of these events involve EFN-4/Ephrin, MAB-20/semaphorin-2a, and SAX-3/Robo. Once cells in the primordium align along a common axis and in the correct order, cells at the anterior end rotate clockwise around the axis of the intestine. The anterior rotation appears to align segments of the developing lumen into a continuous structure, and requires the secreted ligand UNC-6/netrin, the receptor UNC-40/DCC, and an interacting protein called MADD-2. Previous studies showed that rotation requires a second round of LIN-12/Notch signaling in cells on the right side of the primordium, and we show that MADD-2-GFP appears to be downregulated in those cells.

## Author Summary

This report uses the intestine of the nematode *C*. *elegans* as a model system to address how progenitor cells form a three-dimensional organ. The fully formed intestine is a cylindrical tube of only 20 epithelial cells, and all of these cells are descendants of a single cell, the E blastomere. The E descendants form a primordium that changes shape over time as different E descendants divide and move. Cells in the primordium must continually adhere to each other during these movements to maintain the integrity of the primordium. Here, we generated a 3D graphic reconstruction of the developing intestine in order to analyze these events. We found that the cell movements are highly reproducible, suggesting that they are programmed by asymmetric gene expression in the primordium. In particular, we found that the conserved receptor LIN-12/Notch appears to modulate left-right adhesion in the primordium, leading to the asymmetric packing of cells. One of the most remarkable events in intestinal morphogenesis is the circumferential rotation of a subset of cells. We found that rotation appears to have a role in aligning the developing lumen of the intestine, and involves a conserved, UNC-6/netrin signaling pathway that is best known for its roles in the guided growth of neurons.

## Introduction

Epithelial tubes are fundamental components of most animal organs, where they have multiple functions that include the transport of liquids, gases or food [1, 2]. The *C*. *elegans* digestive tract provides a simple genetic model system for studying epithelial cell polarization and tube morphogenesis [3–7]. The digestive tract consists of three linked epithelial tubes, the pharynx, valve, and intestine. Like other organs in *C*. *elegans*, these tubes are built from remarkably few cells; the pharynx contains 80 cells, the valve contains 6 cells, and the intestine contains 20 cells [8]. The various tubes have a circumference of no more than 9 cells, and as few as one, donut-shaped cell. *C*. *elegans* is able to form what are essentially micro-organs because it is able to control the positions and three-dimensional shapes of individual cells, creating differences between adjacent, or even sister, cells. This control is most obvious in the pharynx, which contains several different types of cells organized with distinct and reproducible symmetries. Pharyngeal morphogenesis involves an intermediate, cyst stage, where cells have completed division, developed apicobasal polarity, and become wedge shaped. The cyst transforms into a tube as cells move into their final, cell-type specific positions, by rotating clockwise or counterclockwise around the central axis [9]. Similar rotations occur in the development of the valve and intestine tubes, but the cues that guide the cell rotations are not known.

*C*. *elegans* has several genes that function in directed cell movements and that are conserved in higher animals. For example, the anterior migration of muscle processes requires the Eph receptor VAB-1 and an Ephrin, EFN-1 [10, 11]. The dorsal-ventral guidance of some neurons is thought to involve a ventral gradient of the ligand UNC-6/Netrin [12–14]. Movement toward UNC-6/Netrin can be mediated, in part, by the receptor UNC-40, a homolog of DCC (Deleted in Colorectal Cancer), and movement away from UNC-6/Netrin can be mediated by the receptor UNC-5 acting with UNC-40/DCC [15]. A reciprocal, dorsal gradient of SLT-1/Slit is thought to repel some axons through its receptor, SAX-3/Robo [16]. Some of these same genes have developmental roles in other types of cells, such as in the dorsal-ventral extensions of muscles [17, 18].

**Fig 1:**
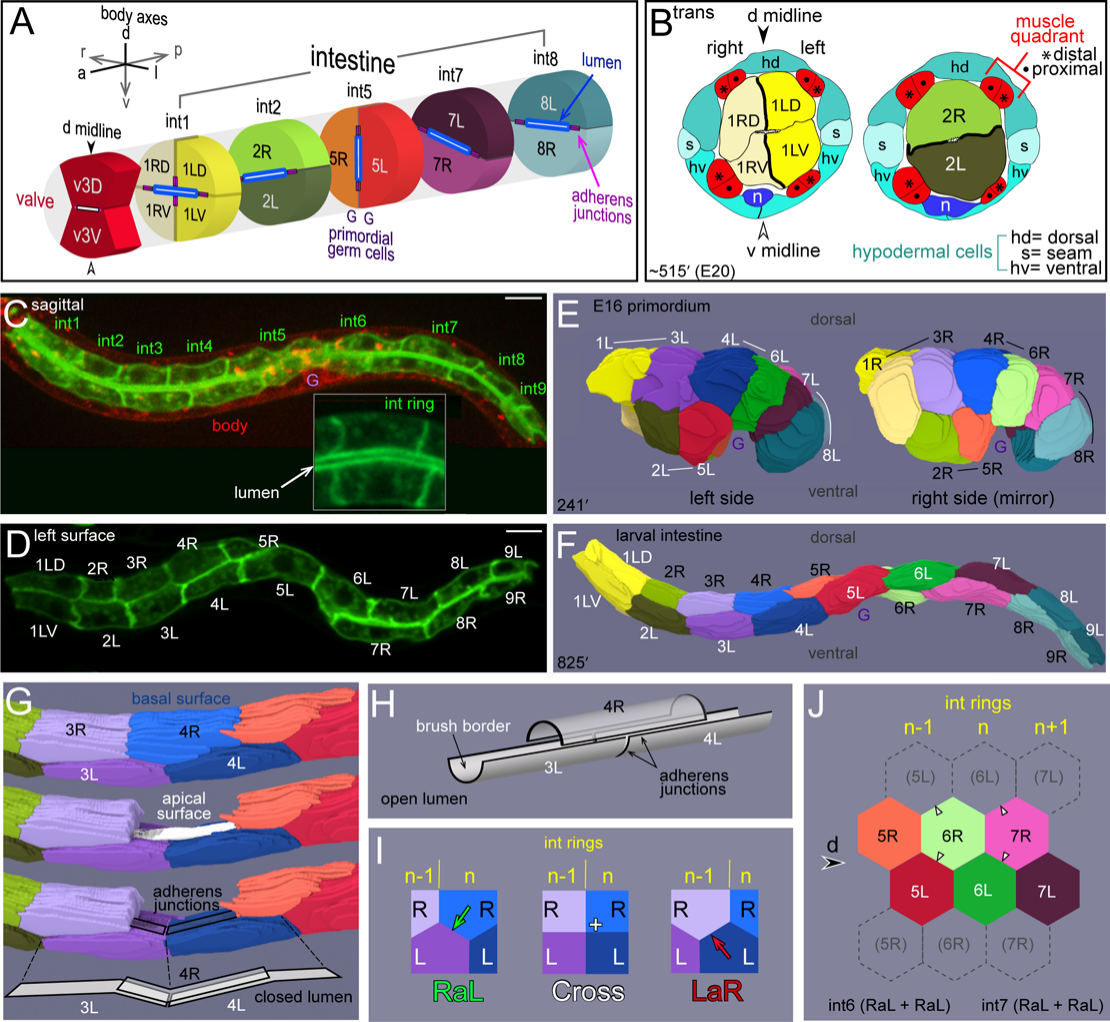
Intestine anatomy. (A) Schematic diagram of selected intestinal rings (int rings) and the terminal valve ring in a newly hatched larva. R (right) and L (left) designate the positions of the labeled cells at the E16 stage. The int rings are color coded with different hues; R and L cells within each int ring are colored with different saturations of the same hue. The arrows shown for the body axes indicate the direction of view for all optical stacks. For example, views of optical sections through either the left or right side of the primordium will be displayed with anterior to the left and dorsal top. (B) Diagram of transverse sections through a single embryo at about 515 minutes, showing cell and tissue boundaries. The drawings are tracings from serial-section electron micrographs available at http://www.wormimage.org/imageList.php?wormName=embryo_RDD. Each of the four muscle quadrants consists of a longitudinal row of proximal muscles (with respect to the midline) and a single row of distal muscles. The quadrants also include several muscle processes that are not included in the diagram. (C and D) Images of live, newly hatched larvae expressing transgenic reporters for intestinal cells (green) and for all membranes (red). Panel C is an optical plane through the center of the intestine, showing the closed lumen, and panel D is an optical plane near the surface of the intestine. The inset in panel C shows a single int ring; the membranes around the lumen appear thicker than the lateral membranes because of the numerous microvilli in the brush border. (E and F) Images from 3D reconstructions of the E16 primordium (panel E; S1 Video) and of the intestine in a newly hatched larva (panel F; S2 Video). The right side of the primordium in panel E is shown as a mirror-right image for comparison with the left side view, and to match the orientation of optical sections presented throughout the paper. (G) High magnification view of the int3 and int4 rings from the reconstruction as in panel F. The 4R cell is removed to show internal cell surfaces, the intestine lumen, and adherens junctions flanking the lumen. Note that the reconstructed apical surface is flattened or closed; see also S4A Fig. (H) Diagram of the intestinal lumen after it opens. (I) Diagram of the basal surfaces of intestinal cells showing possible diagonal contacts between the lateral faces of four cells in adjacent int rings; RaL = Right to anterior Left, LaR = Left to anterior Right. (J) Diagram of the basal surface of a tube of hexagonally packed intestinal cells, cut along the ventral midline and unrolled. Phantom cells (dashed lines) are included to show the complete pattern of diagonal contacts. Panels C and D show strain JJ2360. Bars = 5 microns.

In this report, we examine cell positioning during intestinal morphogenesis. The intestine in a newly hatched larva is a narrow, twisted tube [8]. The epithelial cells are organized into an anterior-posterior series of transverse rows, called intestine rings or int rings. The first int ring (int1) contains four cells, and all other int rings contain two cells each (Fig 1A and 1D). Cells in a given int ring arise from symmetrical cell lineages and have similar shapes, but can differ from cells in other rings. For example, the int5 cells associate specifically with the two primordial germ cells (Fig 1A), and only cells in the int3-int9 rings express the PHO-1 acid phosphatase [19].

All 20 intestinal cells are derived from a single cell in the 8-cell embryo, called the E blastomere; the molecular specification of the E blastomere has been studied extensively and involves multiple pathways [5]. An isolated, cultured E blastomere divides to form a rounded cyst of polarized cells, but never forms a tube [6]. In the intact embryo, the E descendants initially form a primordium, and each stage of the primordium is named according to the number of E descendants; for example, E8 or E16 [6, 8]. Apicobasal polarity is visible by the E16 stage, when the primordium contains two distinct layers of cells. Specific cells intercalate orthogonal to the central axis, such that all cells eventually align in two, longitudinal rows; these movements establish the basic form of the tube. Next, a subset of the anterior int rings rotate around the lumen clockwise, as viewed from the front of the animal [8, 20, 21]. The purpose of these rotations, collectively called intestinal twist, is not understood, but the rotations require the LIN-12/Notch signaling pathway [21, 22]. LIN-12 signaling occurs twice in the intestinal primordium. Between the E4 and early E8 stages, the receptor LIN-12 is expressed in all intestinal cells. Ligand-expressing, non-intestinal cells contact the left side of the primordium and activate the expression of REF-1, a bHLH transcription factor that is a direct target of LIN-12 [22, 23]. At least one function of REF-1 is to downregulate LIN-12 in the left cells, apparently through the transcriptional corepressor UNC-37/Groucho. Thus, only right cells continue to express LIN-12 at the E16 stage, when a second LIN-12/Notch interaction triggers a new round of REF-1 expression [22]. Mutant analysis has shown that the second interaction is required for twist, suggesting that at least one function of the first interaction is to generate left-right asymmetry for the second interaction [21].

Here, we used confocal microscopy to visualize intestinal cell shapes during morphogenesis. We generated 3D reconstructions of the developing primordium, and used the reconstructed stages to assemble a movie of intestinal morphogenesis. The color-coded movie and 3D reconstructions facilitate visual tracking of individual cells, and provide several new insights into intestinal morphogenesis. We show that individual intestinal cells are positioned with a reproducible choreography that implies early anterior-posterior and left-right differentiation between cells. We show that LIN-12/Notch signaling is required for left-right asymmetry in intestinal cell contacts, hours before the known requirement for LIN-12/Notch in intestinal twist. Finally, we show that several genes with roles in axon guidance are important for the circumferential rotations of intestinal cells; we provide evidence that the rotations function to align the lumen, which forms piecemeal in the individual int rings.

## Results

### Anatomy and nomenclature

The anterior end of the intestine is attached to the pharynx by a small, connecting tube called the valve (Fig 1A). The most posterior ring of valve cells contains a dorsal cell called v3D (valve ring 3 Dorsal) and a ventral cell called v3V (valve ring 3 Ventral). The intestine is flanked by a ventral nerve cord, four muscle groups (quadrants), and by a single layer of epithelial (hypodermal) cells (Fig 1B). Transverse views of the body show five hypodermal cells, although the dorsal “cell” is a syncytium created by the fusion of individual dorsal hypodermal cells.

The description of cell movements during intestinal morphogenesis is complicated by conventional, anatomical names for cells that indicate only lineage origins or final positions [8]; different problems arise with an alternative system we proposed that numbers cells solely according to their initial positions [6]. Here, we use a hybrid nomenclature that labels cells according to their int ring number, and as either R (Right) or L (Left) depending on their positions in the E16 primordium. The E16 primordium is a convenient reference point, as it has a simple, bilateral symmetry, and contains most of the final 20 intestinal cells. For example, 2R is a right cell in the E16 primordium that later forms part of the int2 ring (Fig 1A). This nomenclature emphasizes that each int ring begins as one R cell and one L cell. The R and L cells in the int1 ring undergo an additional, dorsal-ventral division to form a 4-cell ring. Cells in the int8 ring also undergo an additional division, but this division is anterior-posterior and creates a new, int9 ring. We observed infrequent, anterior-posterior divisions of 7R and/or 7L, consistent with previous results that intestines in newly hatched larvae occasionally have more than 20 cells [20].

We used confocal microscopy to visualize intestinal cell shapes and cell contacts in embryos and in live, newly hatched larvae (Fig 1C and 1D). In most experiments, embryos/larvae expressed three fluorescent reporters for general membranes, intestine-specific membranes, and for pharyngeal nuclei; the pharyngeal nuclei are included for spatial reference (Materials and Methods). Cell contours were traced and used for three-dimensional, graphic reconstructions of the developing primordium (Fig 1E; S1 Video) and of the larval intestine (Fig 1F, S2 Video). Inspection of the color-coded reconstruction illustrates some of the cell rearrangements that occur between the E16 stage and hatching. For example, 2R is a ventral cell in the E16 primordium (Fig 1E), but becomes a dorsal cell in the fully formed intestine (Fig 1F). Similarly, 4L and 6L are adjacent, sister cells in the E16 primordium, but are separated by 5L in the larval intestine. In essence, intestinal morphogenesis creates a longitudinal row of R cells that twists around a longitudinal row of L cells (Fig 1F).

Schematic diagrams of the intestine typically depict int rings as smooth discs (Fig 1A and [5, 8]). However, cells within an int ring are offset, like bricks in a wall, and the actual int rings have jagged boundaries (Fig 1C and 1F). The offset extends to the interior, apical surfaces of cells, and results in a complex, ladder-like appearance of adherens junctions (Fig 1G and 1H). Rotating the 3D reconstruction reveals that the peripheral, basal surfaces of the intestinal cells are predominantly hexagonal (S2 Video). To describe the pattern of cell packing, we score the diagonal contacts between the lateral surfaces of cells in two, adjacent int rings (Fig 1I). If the R cell in a given ring, n, contacts the L cell in the anterior, n-1 ring, we term the contact RaL (R to anterior L). If the L cell in the n ring contacts the R cell in the n-1 ring, we term the contact LaR (L to anterior R). If all four cells meet at a common vertex, we term the pattern Cross. Because the intestine is a narrow tube, rather than a flat sheet, additional diagonal contacts are possible between the same four cells (Fig 1J). For example, 6R makes diagonal contacts with two different faces of 5L; both of these are RaL contacts. Thus, the complete description of diagonal contacts between adjacent int rings can involve a combination of RaL, LaR, and Cross contacts. We found that cells in the int5-int8 rings are almost exclusively hexagonal in the larval intestine, and that nearly all diagonal contacts are RaL+ RaL (Fig 1J; Table 1). The int2-4 rings can contain non-hexagonal cells, and can have both LaR and RaL diagonal contacts (Table 1). For example, int2 always makes equal RaL and LaR contacts with the 4-cell int1 ring; 2R to 1LD (RaL) and 2L to 1RV (LaR) (Fig 1A, Table 1). We refer to the general predominance of RaL contacts in the larval intestine as RaL asymmetry.

**Table 1:** Diagonal RaL, LaR, and Cross contacts in intestinal cells at hatching.

	Diagonal contacts (%)						
int ring	RaL+RaL	LaR+LaR	RaL+LaR	RaL+C	LaR+C	C+C	n
int2	0	0	100				35
int3	12	3	61	12	9	3	33
int4	66	3	31				32
int5	97	0	3				34
int6	100	0	0				35
int7	100	0	0				35
int8	100	0	0				35

### Intestinal morphogenesis: Oriented cell divisions and *vang-1*-dependent cell repositioning create a rectilinear E8 primordium

**Fig 2:**
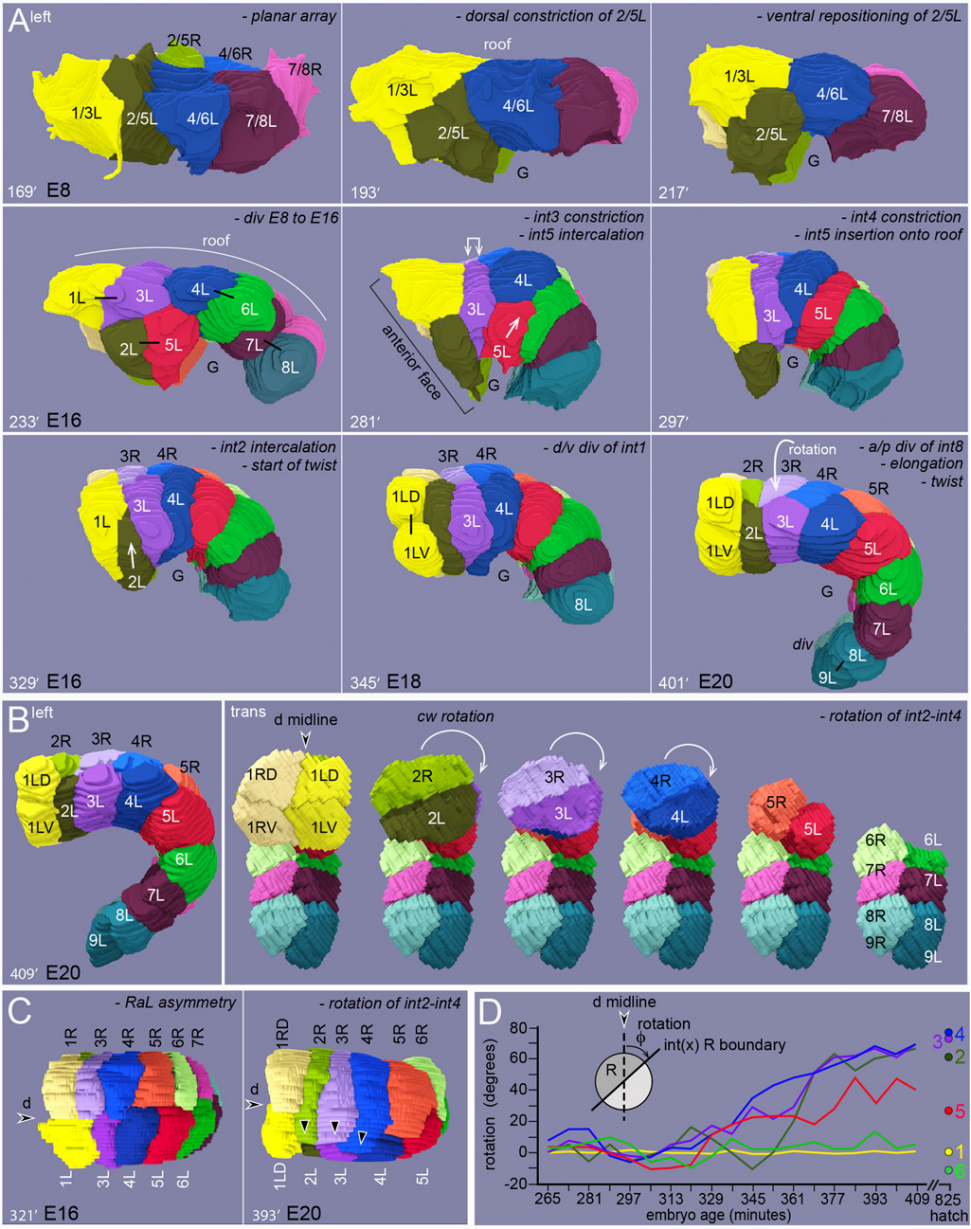
Summary of intestinal morphogenesis. (A-C) Key frames from the movie of the reconstructed primordium (S1 Video). Major developmental events are summarized for each panel (italics). (A) Left side of the primordium from 169–401 minutes. The dorsal side of the primordium is up and anterior is to the left. (B) The panel at left shows the left side of the primordium at 409 minutes. The right panels show the primordium rotated face on, with successive int rings removed to show rotation of the int2-4 rings. (C) Dorsal views of the primordium at 321 and 393 minutes. (D) Quantitation of R cell rotation in the reconstruction, measured in degrees from the dorsal midline. The circumference of the primordium is about 47 microns, so the rotation of int4 averages about 0.1 μm/min.

Our 3D reconstruction of the developing primordium was generated from a single embryo, imaged at 8-minute intervals between the E8 and E20 stages (S1 Video). Additional, partial reconstructions were generated from other embryos, and these showed essentially no differences from the reference reconstruction except for variation in the pattern of int2 intercalation (see below). Selected timepoints from the reconstruction that represent key developmental events are shown in Fig 2A–2C. For clarity in presentation, all developmental times are referenced to the reconstruction (S1 Video) by matching the first timepoint in an image sequence to the closest timepoint in the reconstruction; subsequent time intervals are as indicated (see Materials and Methods). We present our findings on cell shapes and contacts within a general outline of intestinal morphogenesis, but only briefly review events that have been described in detail elsewhere [5, 6].

**Fig 3:**
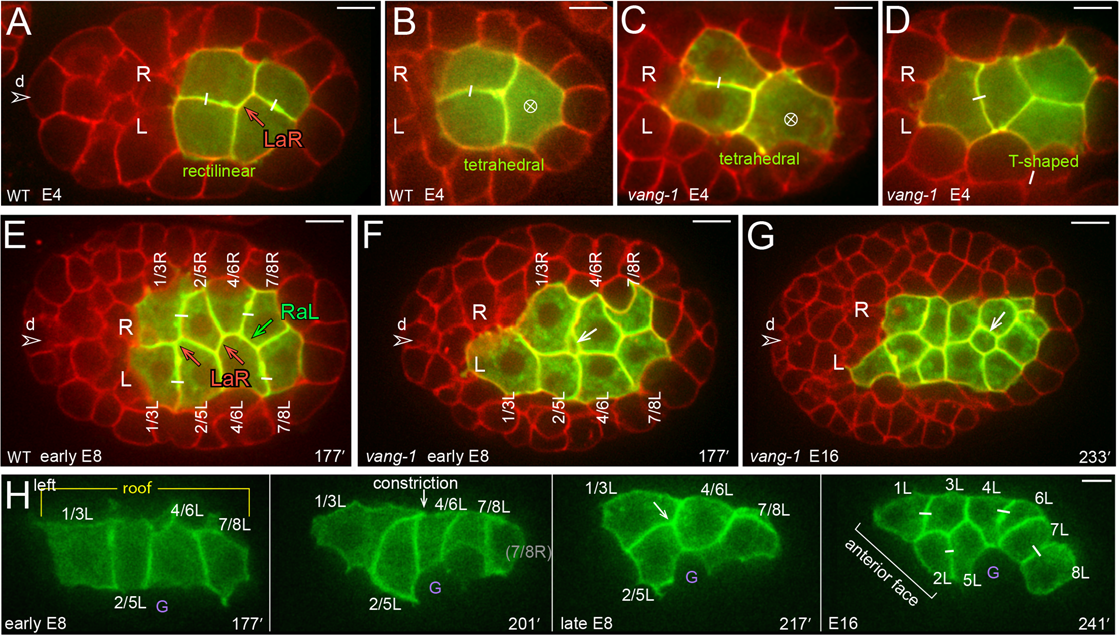
Cell repositioning between the E4 and E16 stages. (A-G) Each panel shows a horizontal, optical plane through the intestine primordium (green) of a live embryo. Small white bars link sister cells born from divisions within the plane, and a white, circled X indicates a division perpendicular to the optical plane. The wild-type E4 primordium is either rectilinear (panel A), tetrahedral (panel B) or T-shaped. Similarly, the *vang-1* E4 primordium can be rectilinear, tetrahedral (panel C), or T-shaped (panel D). An example of a LaR diagonal contact is shown in panel A. (E) Wild-type E8 primordium. The cells have formed a plane, and show one RaL contact in addition to two LaR contacts. (F) *vang-1* mutant E8 primordium with non-planar cells (white arrow). (G) *vang-1* mutant E16 primordium with misplaced cells (white arrow); compare with dorsal view of the wild-type E16 primordium in S1 Video. (H) Image sequence showing the left side of a wild-type primordium between the E8 and E16 stages. The primordium is bilaterally symmetrical; the right side of a similar wild-type primordium is shown in S3 Video. Note that the entire primordium shifts dorsally (up) over time. Panels = A,B,E [strain JJ2360], B,C,D,F,G [strain JJ2515]. Bars = 5 microns in A-G, 5 microns in H.

The E8 primordium is a relatively flat, rectangular plane of four R cells and four L cells (Fig 2A). In most embryos, the planar shape of the E8 primordium appears to result from the oriented divisions of the E blastomere and its descendants [6]. The E blastomere divides anterior-posterior, the E2 cells divide right-left (Fig 3A), and the E4 cells divide anterior-posterior (63%, n = 60; Fig 3E). However, previous studies noted variability in the positions of some E4 cells in wild-type embryos [24], and we found that the E4 primordium can be tetrahedral and non-planar, or planar but shaped like a T (27%, n = 60; Fig 3B). Nevertheless, each of these embryos develops a planar E8 primordium (100%, n = 60). Thus, a mechanism must exist to reposition variant cells before or during the E8 stage.

Previous studies showed that VANG-1 has a role in intestinal development; VANG-1 is the only *C*. *elegans* homolog of Strabismus/Van Gogh, a component of the planar cell polarity (PCP) pathway [25]. Those studies showed that 32% of *vang-*1 mutants have abnormal intestines, but the origin of the defects was unknown. We found that the E4 primordium in *vang-1* mutant embryos exhibited variability in shape that was similar to the variability observed in wild-type embryos. Like the wild-type primordium, the *vang-1* E4 primordium could be rectilinear and planar (41%, n = 85), or have a variant shape (59%; Fig 3C and 3D). By contrast with wild-type embryos, the *vang-1* mutant embryos failed to reposition the variant cells, resulting in an E8 primordium that remained misshapen and non-planar (39%, n = 85 embryos; Fig 3F). Nearly all of the *vang-1* embryos with a misshapen E8 primordium developed an abnormal E16 primordium, with defects in int ring organization that were similar to defects reported previously (Fig 3G). These results suggest that oriented cell divisions act in concert with VANG-1-mediated cell positioning to align the wild-type E8 cells into a plane.

The planar E8 primordium becomes a bilayered, E16 primordium [6]. Previous studies that used differential interference contrast (DIC) microscopy to follow nuclei suggested that diagonal divisions of the E8 cells might create the layering [6]. However, we found that the E8 cells reposition before cell division (Fig 3H and S1 Video). Repositioning begins when the second, transverse row of E8 cells (2/5L and 2/5R) constrict their dorsal surfaces, shifting their cell bodies ventrally. At about the same time, flanking cells in the first and third rows extend thin, sheet-like processes that close together over the second row. After closing, the dorsal contact between the first and third rows increases and further displaces the second row cells ventrally. Thus, when the E8 cells divide to form the E16 primordium, the daughters of the second row cells are ventral to other cells (Fig 3H).

The primordium shifts dorsally during the E8 stage (Fig 3H), suggesting that the repositioning of the second row cells could result from forces that restrict their dorsal movement. For example, the primordial germ cells attach to the ventral surface of the primordium, where they “hitchhike” into the body interior [26]. We used a laser microbeam to kill the parent of primordial germ cells (the P4 blastomere), but observed normal, ventral repositioning of the second row of E8 cells in each of five operated embryos. Similarly, we observed normal repositioning of the second row cells after ablating the precursor of the hypodermal cells that lie above the E8 primordium (the C blastomere, 8/8 operated embryos). These results suggest that the ventral repositioning of the E8 cells results from forces generated within the primordium.

### Cell intercalations align bilayered E16 cells to a common axis

**Fig 4:**
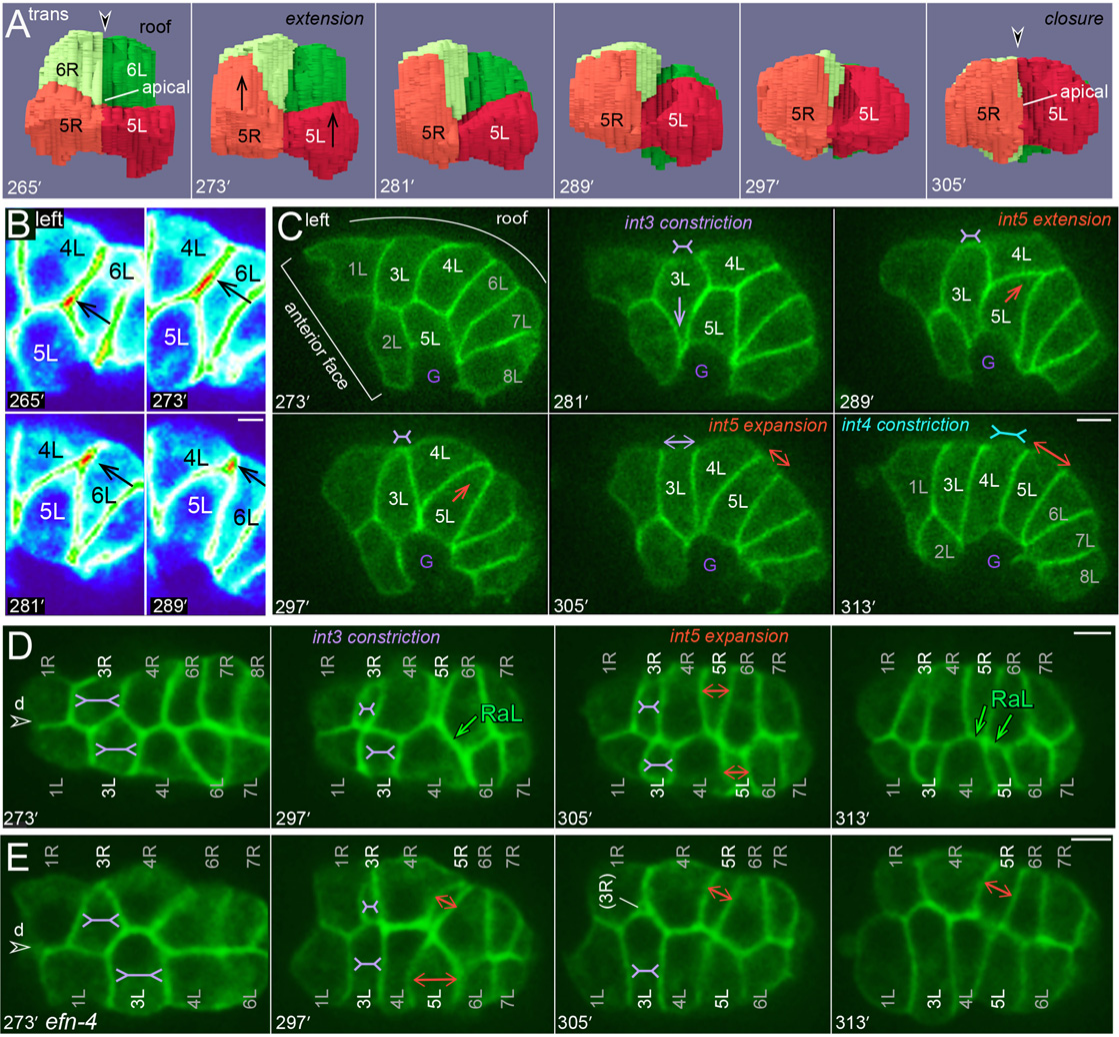
Intercalation/closure of the int5 ring. (A) Transverse view of the reconstructed primordium showing the int5 and int6 cells during int5 intercalation/closure. The nascent apical surface is indicated at 265 and 305 minutes [6]. (B) Heat-map representation of GFP fluorescence intensity (red = high) at the leading edge of 5L during intercalation. The increased signal at the leading edge is seen with both of the membrane reporters used in this study, and precedes cytoplasmic flow into the same region (see S3 Video). We interpret the increased signal as resulting from the transient presence of four membranes (protrusion plus flanking cells), relative to the signal from two membranes at other cell contacts between intestinal cells. (C) Image sequence showing the left side of the E16 primordium during int5 intercalation. The dorsal surface of 3L constricts (purple, inverted double arrow) as 5L begins to shift dorsally. After reaching the roof, 5L expands longitudinally (orange double arrow) as the dorsal surface of 4L constricts (blue, inverted double arrow); see also S3 Video. (D, E) Image sequences of a wild-type embryo (D) and an *efn-4* mutant embryo (E) during int5 intercalation, imaged at an optical plane near the roof of the primordium. Note the constriction of 3L and 3R in both embryos. In the *efn-4* mutant, 3R partially separates from 3L, and detaches entirely from the roof of the primordium; 3R rejoined the roof 64 minutes later. Panels = B,C,D [JJ2360]. E, [JJ2486]. Bars = 5 microns.

**Fig 5:**
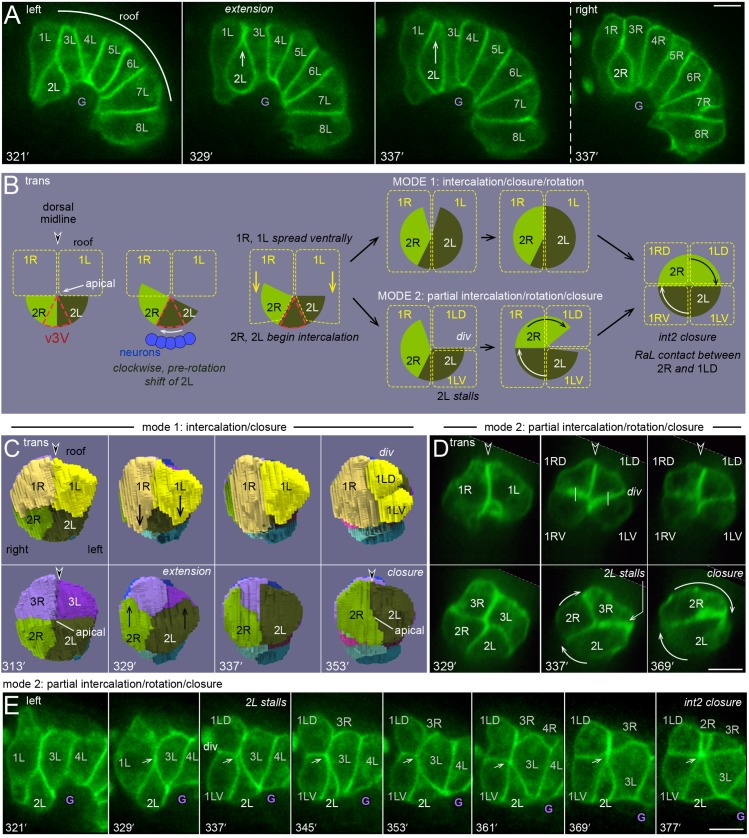
Intercalation/closure of the int2 ring. (A) Left lateral view of the E16 primordium during int2 intercalation/closure; note that cells flanking 2L do not appear to undergo dorsal constriction. In this embryo, 2R intercalations lags 2L intercalation, but 2L does not cross the dorsal midline to connect with 2R. (B) Diagram of the two modes of int2 intercalation/closure. Before intercalation, 2L shifts clockwise across ventral neurons and behind the ventral midline valve cell, v3V (red dashed outline). The int1 cells spread ventrally prior to division, across the anterior face of the int2 cells. The int2 cells either intercalate to close the ring and then rotate clockwise (mode 1), or 2L stalls and 2R completes the ring by rotating across the dorsal midline (mode 2). (C) Transverse view of int2 intercalation, mode 1, from the 3D reconstruction. (D) Transverse view of int2 intercalation, mode 2. The dorsal extension of 2L stopped when 1L divided, and the ring was closed by 2R. (E) Left side view of int2 intercalation, mode 2. Here, 2L extends dorsally between 321 and 329 minutes, at which point 1L divides and 2L stalls. Int2 closure occurs only after 2R reaches 2L on the left side. Note that the elapsed time from extension to closure is 58 minutes in this embryo, compared to 24 minutes for mode1 in Fig 4C. Panels = A,D,E [JJ2360]. Bars = 5 microns.

The ventral repositioning of the second row of E8 cells, and the subsequent E8 cell divisions, expand the anterior face of the E16 primordium such that it covers the entire posterior surface of the adjacent pharynx/valve primordium (Figs 2A and 3H) [9]. It is not known whether ventral repositioning is essential to create the final, tubular shape of the intestine, but the expanded face appears critical for the proper polarization of the pharynx and valve cells (see Discussion). After the pharyngeal and valve cells polarize, the face of the intestinal primordium reduces in size as the int5 cells (S3 Video), and subsequently the int2 cells (S4 Video), intercalate and re-align in a common axis with other E16 cells. The int5 and int2 intercalations begin at about 273 minutes and 329 minutes, respectively (Figs 4A and 5A). Because previous studies showed that intestinal cells develop apicobasal polarity by about 257 minutes [6], both intercalations involve oriented movements by polarized cells. In essence, the intercalating cells complete their int rings by closing together around their nascent apical surfaces. The intercalation paths appear invariant, with the int5 cells extending between the int4 and int6 rings, and the int2 cells extending between the int1 and int3 rings. The intercalations are led by thin, lateral protrusions that are visible by confocal microscopy as an increase in local, membrane fluorescence (Fig 4B; see also [9]). As the leading poles of the intercalating cells extend, the trailing poles become rounded and then flattened (Figs 4B and 5A); we presume the rounding reflects de-adhesion/contraction of the trailing pole.

The intercalating int5 cells do not appear to simply force aside flanking cells. Instead, the neighboring cells undergo invariant changes in cell shape that likely facilitate int5 intercalation (S3 Video). First, the dorsal surfaces of the right and left int3 cells constrict markedly (Fig 4C and 4D); this constriction appears to shift int3 cytoplasm ventrally, before most of the int5 cytoplasm shifts dorsally. After the int5 protrusions reach and close together at the roof, there is an apparent dorsal constriction of the int4 cells; this latter constriction appears to shift int4 cytoplasm ventrally, allowing the int5 cells to expand longitudinally onto the roof (Fig 4C).

The intercalation/closure of the int2 cells is considerably more variable than for the int5 cells; it can span 24 to over 48 minutes, and does not show a consistent coupling with changes in the shapes of flanking cells (Figs 5A and S1; S4 Video). The int2 ring intercalates and closes using one of two general modes (diagrammed in Fig 5B; examples of further variations within each mode are shown in S1 Fig). The first mode closely resembles int5 intercalation/closure, and occurs in about 27% of wild-type embryos (n = 7/26). Here, both of the int2 cells extend to, and close together at, the dorsal midline (Fig 5C). In the second mode, the 2L cell extends dorsally for variable distances, but stops or stalls before reaching the dorsal midline; the stalling nearly always occurs when the adjacent 1L cell divides (Fig 5D and 5E). 2L does not resume intercalation, and 2R closes the ring by rotating clockwise, past the dorsal midline, until it reaches 2L on the left side of the primordium (Fig 5B, 5D and 5E). Although 2R intercalation can also stall when the adjacent 1R cell divides, 2R always resumes intercalation/rotation. The longitudinal expansion of the int2 ring onto the roof can occur considerably after closure, by contrast with the immediate expansion of the int5 ring. Instead, int2 expansion occurs gradually, when the intestine and the body of the embryo begin to elongate (Fig 5E; S1 and S3 Videos).

### RaL asymmetry involves Notch-dependent, polarized cell rearrangements

**Fig 6:**
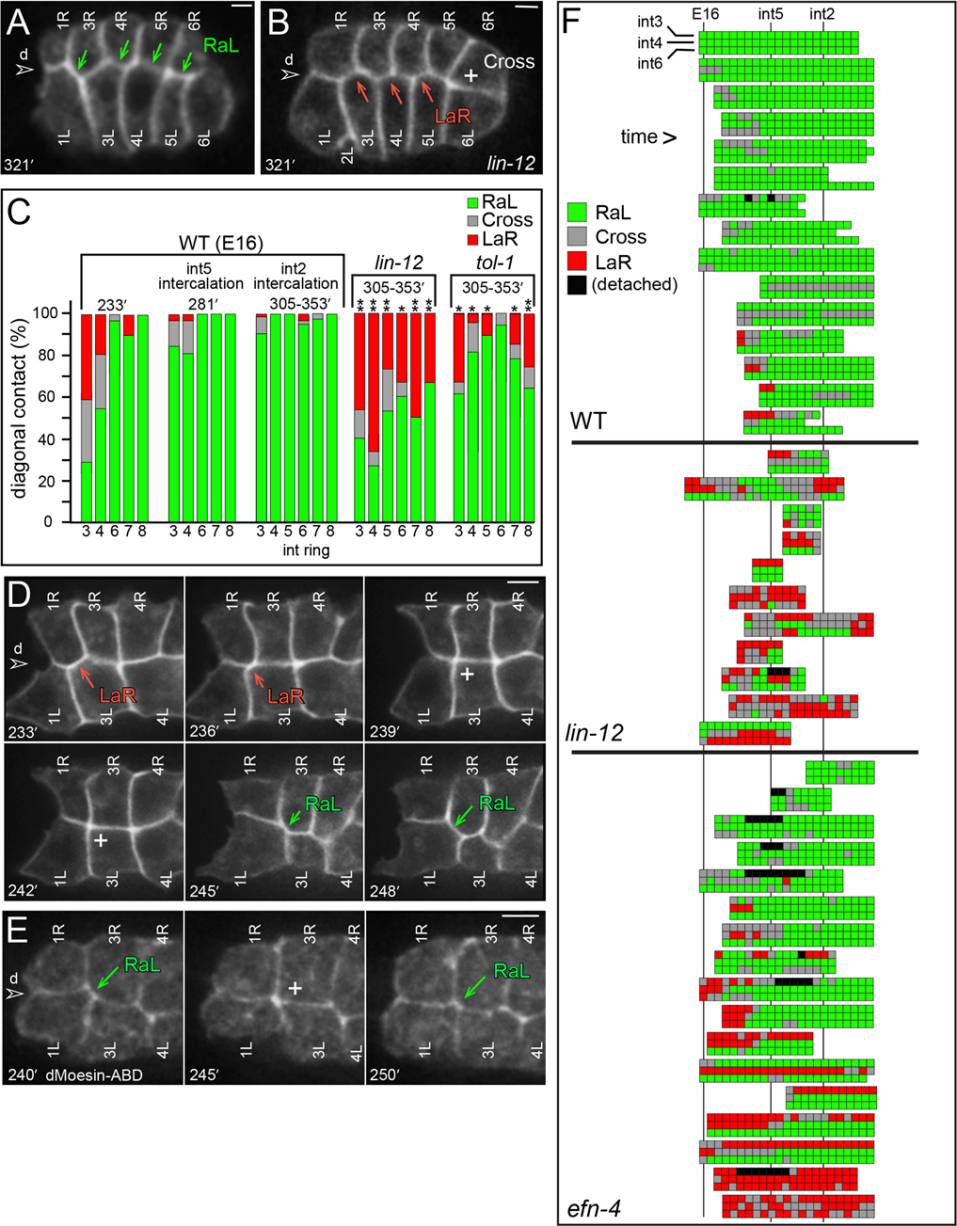
Diagonal contacts between int rings. (A,B) Dorsal roof of the E16 primordium in a wild-type embryo (A) and a *lin-12(n941)* mutant embryo (B), showing diagonal contacts between int rings. (C) Quantitation of diagonal contacts between int rings at the indicated stages. One star indicates P < .05, two stars indicate P<5E-04; Fisher’s exact test (WT = 29–36 contacts, *lin-12* = 15–23, *tol-1* = 11–28). (D) Image sequence showing dynamic diagonal contacts between int rings. (F) Image sequence showing filamentous actin localization (GFP::dMoeABD) as the diagonal contact from int3 to int1 changes from RaL, to Cross, and back to RaL. (F) Diagram showing changes in diagonal contacts over time for the indicated rows in single embryos. Each box represents an 8-minute interval; boxes are aligned vertically with respect to the finish of int5 intercalation and the beginning of int2 intercalation. Black boxes are events where an R and L cell within a ring partially separated and/or detached from the roof of the primordium (see Fig 4E). Panels = A,D [JJ2360], B [JJ2492], E [*nnIs*[*unc-119(+) pie-1 promoter*::*gfp*::*Dm-moesin*^*437-578*^[76]]. Bar = 2.5 microns.

We wanted to determine how int rings develop the predominately RaL pattern of cell packing observed in the fully formed intestine (Fig 1F). The first diagonal contacts occur in the E4 primordium. In embryos with a planar, rectilinear E4 primordium, the contacts are either LaR (79%) or Cross (21%, n = 28 embryos; Fig 3A). Some diagonal contacts begin to transition to RaL during the E8 stage (Fig 3E and Table 2), and most become RaL within one hour of the E16 stage (Fig 6A and 6C; S5 Video). We discovered that the transition to RaL contacts requires the LIN-12/Notch signaling pathway; nearly half of *lin-12(n941)* null mutant embryos examined at the E16 stage have multiple LaR or Cross contacts (Fig 6B and 6C).

**Table 2:** Diagonal contacts in the E8 primordium.

	Diagonal contacts (%)							
	Early E8*				Late E8**			
int ring	RaL	LaR	C	n	RaL	LaR	C	n
int2/5	0	65	35	23	-	-	-	
int4/6	4	91	5	23	42	42	16	26
int7/8	26	48	26	23	92	4	4	26

*before the ventral repositioning of 2/5R and L. Note that before repositioning the diagonal contacts scored for int4/6 are with the anterior ring, int2/5.

**After the ventral repositioning of 2/5R and L. Here, the diagonal contacts scored for int4/6 are with int1/3.

Live imaging showed that the transition to RaL involves dynamic neighbor exchanges (Fig 6D and 6F). For example, a LaR contact can transition to Cross, remain for several minutes, and then return to LaR or convert to RaL. Filamentous actin, as indicated by dMoesin-ABD localization, appears to increase transiently in Cross contacts (Fig 6E), suggesting that the Cross configuration is created by localized cell constrictions. Most wild-type int rings maintain RaL contacts after the E16 stage, although contacts in the in2-4 rings are modified by rotation (Fig 6F and see below). Intercalation of the int5 cells breaks a RaL contact from 6R to 4L (Fig 4D at 297 minutes), but that contact is soon replaced by new RaL contacts from 5R to 4L, and from 6R to 5L (Fig 4D at 313 minutes). Similarly, the RaL contact from 3R to 1L breaks during int2 intercalation (S1 Fig). By contrast with wild-type embryos, the int rings in *lin-12(n941)* mutant embryos continue to switch between LaR and RaL contacts throughout the E16 stage (Fig 6F).

The above analysis scored diagonal contacts at a single optical plane near the roof of the primordium (the basal surface). However, inspection of complete optical stacks showed that some rings with a RaL contact near the roof could also have an internal LaR contact (S2A and S2B Fig). We used the 3D reconstruction to approximate the entire surfaces areas engaged in RaL or LaR contacts over time (S2D and S2E Fig). These measurements show that the transition to RaL contact involves the entire lateral surfaces of the cells, and that the increase in RaL occurs by the relative loss of LaR contact, rather than by an increase in cell volume (S2C Fig).

Recent studies have demonstrated that polarized cell rearrangements in *Drosophila* epithelial cells involve Toll family receptors that are expressed in an overlapping, striped pattern [27]. Toll signaling pathways are best known for their conserved roles in innate immunity, and *C*. *elegans* contains homologs of multiple genes in the innate immunity pathway [28]. Interestingly, mutants lacking TOL-1, the sole *C*. *elegans* Toll-like receptor, have severe developmental defects that are not observed for putative null alleles of the other innate immunity genes [28]. We found that the int rings in *tol-1(nr2013)* mutant embryos had predominately RaL contacts, but had a higher frequency of non-RaL contacts than are observed in wild-type embryos (Fig 6C). Although these results suggest that TOL-1 contributes to intestinal patterning, the mutant embryos have extensive defects in non-intestinal tissues that might preclude normal intestinal development [28].

### Rotation of the int rings

**Fig 7:**
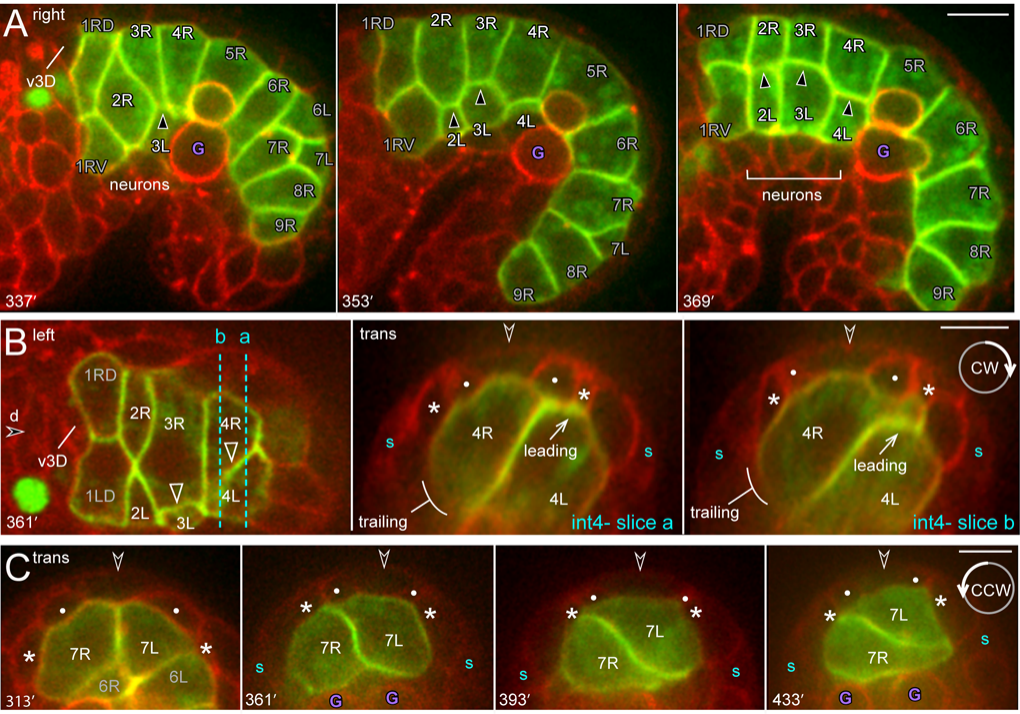
Intestinal twist. (A) Image sequence showing rotation of the int2-int4 rings as viewed from the right side of the primordium; L cells appear in the plane as they rotate clockwise. Here, 3L is the first to rotate, followed by 2L and 4L. The nuclei anterior to the intestine express a transgenic GFP reporter for pharyngeal cells (see Materials and Methods). (B) Oblique, dorsal-left view of the primordium showing 2L intercalation (mode 1), and the start of the clockwise rotation of 4R. The panels at right show transverse projections of the optical stack, viewed at planes a and b. 4R rotation advances in a wave from anterior to posterior, so the projection at plane b later resembles the contemporary plane a. Note that the basal protrusion at the leading pole of 4R increases in thickness as the cell rotates (see also S3A and S3B Fig). Cells outside the primordium are labeled as in Fig 8A. (C) Image sequence showing the counterclockwise rotation of int7. Panels = A-C [JJ2360]. Bar = 5 microns.

The int2-4 rings undergo a clockwise, circumferential rotation, as viewed from the anterior of the primordium (Fig 2B and 2C; S6 Video). Views from the right side of the primordium (Fig 7A) show 2L, 3L, and 4L moving into the optical plane as the complementary R cells move away. The rotation begins at about 337 minutes, and shifts the R/L boundaries at about 0.1–0.2 microns/minute (Fig 2D). Through rotation, the 2R, 3R, and 4R cells become approximately centered below the dorsal midline, and 2L, 3L, and 4L become centered above the ventral midline. The int5 ring does not undergo a comparable rotation, but 5R and 5L shift positions anterior-posterior while remaining in contact with the dorsal midline (Fig 2C). R and L cell movements are tightly coupled within a rotating int ring: As the leading pole of one cell advances (arrowheads in Fig 7A), the trailing pole of the complementary cell retreats with no apparent gaps between cells. By contrast, there is no obvious longitudinal coupling between the rotations of different int rings. For example, a rotating 3R can contact either 2L or 1L, depending on whether int2 has intercalated. Transverse views of rotating cells show that the trailing pole is rounded and that the leading pole extends a thin, basal protrusion over the complementary cell; the protrusion appears to fill with cytoplasm as the cell advances, and a new basal protrusion forms (Figs 7B, S3A and S3B). Cell positions in the intestine of a newly hatched larva show that the posterior L and R cells must also rotate from their initial, bilaterally symmetrical positions (Fig 1F). These events were not studied in detail here, as they occur after the embryo begins to move and roll within the eggshell. However, we found that the initial stages of int7 rotation could be imaged in some embryos, and that the rotation was always counterclockwise, opposite the rotation of the anterior int rings (n = 8 embryos; Fig 7C). The embryos with int7 rotation did not show any rotation of the int8 or int9 cells, indicating that those rotations must occur later in development; the int8 and int9 cells are the last cells to be born in the primordium (Fig 2A at 401 minutes), which might delay their rotations relative to other cells.

**Fig 8:**
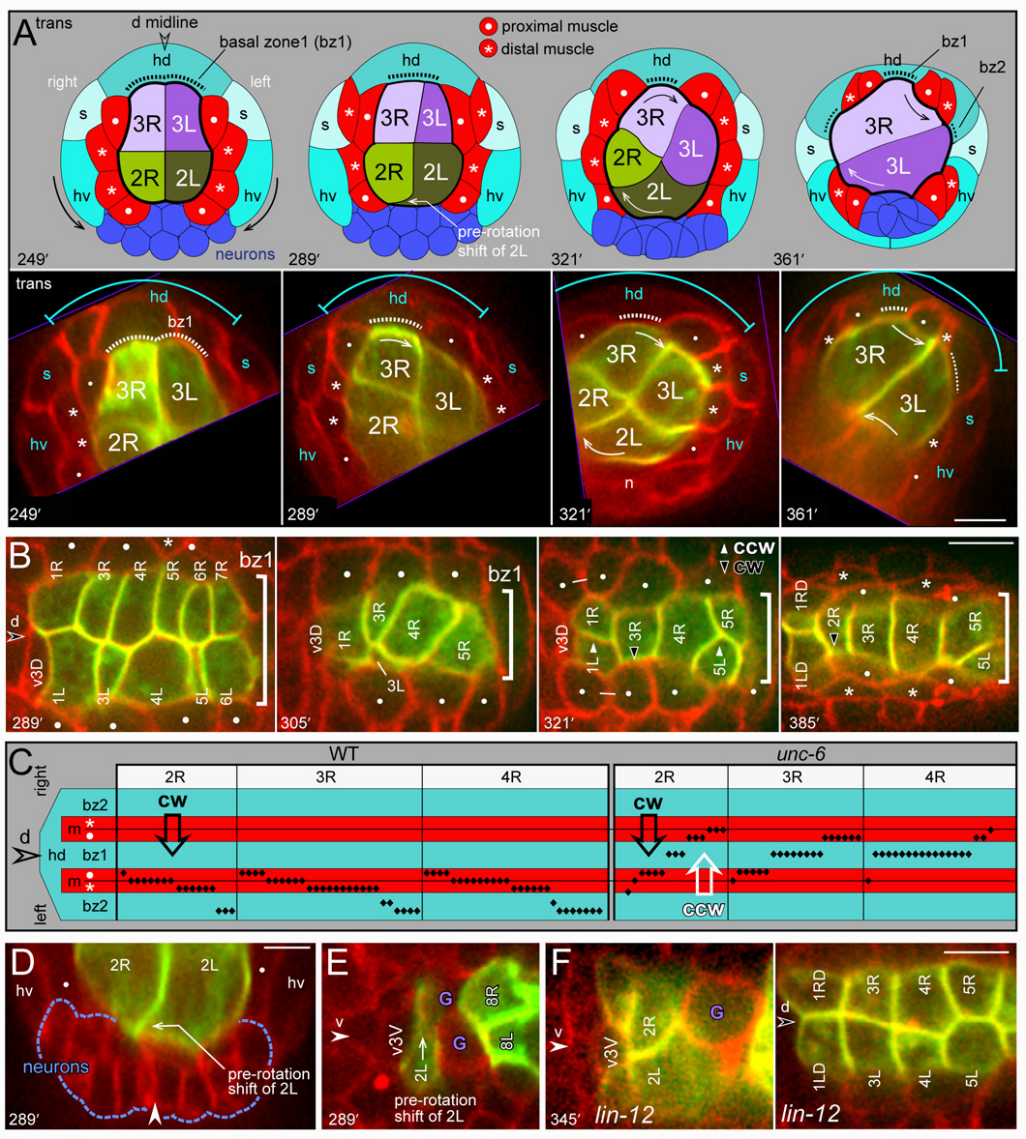
Intestinal twist with respect to surrounding tissues. (A) The top row is a diagram of the rotating cells and surrounding tissues over time; labeling as in Fig 1A and 1B. The bottom row shows representative images of transverse projections through optical stacks of live embryos; the top and bottom of each stack is indicated (purple lines), and the stacks are rotated to such that the midline is vertical. Additional images and details of the surrounding cells are provided in S2 Fig. (B) Image sequence showing the dorsal roof of the primordium over time, imaged at a horizontal plane through the bz1 region (bracket) as defined in panel A. The valve cell v3D invariably rotates counterclockwise under bz1, while R cells typically shift clockwise for variable distances under bz1 (305 minutes). Thereafter 3R, 4R, and later 2R rotate clockwise past bz1, while the int1 and int5 rings shift counterclockwise to center under bz1. (C) Diagram showing the leading poles of R cells (black diamonds) with respect to surrounding, non-intestinal tissues. The data compares wild-type and *unc-6* mutant embryos, scored between about 361–433 minutes (see also Table 3). Note that all wild-type R cells have moved clockwise (cw) past the dorsal midline, but that *unc-6* R cells have moved either clockwise or counterclockwise (ccw). (D,E) Transverse and horizontal planes showing, respectively, the clockwise, pre-rotation shift of 2L over ventral neurons (panel D) and behind v3V (panel E). Note that 2R has relatively little contact with the ventral neurons after the shift. (G) *lin-12* mutant embryo imaged at a ventral horizontal plane through the midline cell v3V (left panel), and at a dorsal horizontal plane through bz1 (right panel). Note that 2L and 2R both contact v3V and ventral neurons, and that the int3 and int4 rings failed to rotate. Panels = A,B, E, F [JJ2360], G [JJ2942]. Bars = 5 microns.

We identified contacts between the intestine primordium and non-intestinal cells before and during rotation of the int2-int4 rings. The contacts are diagrammed in Fig 8A, and changes in the surrounding cells are described in detail in S3 Fig. Briefly, a region below the dorsal hypodermal cells that we term bz1 (basal zone 1) is in direct contact with the primordium. Before int ring rotation, bz1 is a broad region that initially contacts R and L cells (Fig 8B; 249–289 minutes). The dorsal hypodermal cell spreads circumferentially, and bz1 narrows such that it often contacts only, or predominantly, R cells (Fig 8B; 305 minutes). As rotation begins, the 2R,3R, and 4R cells continue clockwise past bz1 and under the left dorsal muscles (Fig 8B; 321 minutes). By contrast, the int1 and int5 rings can rotate slightly counterclockwise at this stage, such that L cells that lost contact with bz1 regain contact (Fig 8B; compare 305 minutes with 321 minutes). During the int ring rotations, the left and right muscle groups that flank the primordium split dorsal-ventral to form the four muscle quadrants (Fig 8A). The dorsal hypodermis inserts between the dorsal and ventral muscle quadrants, and contacts the primordium at a new zone we term bz2 (basal zone 2; Fig 8A at 361 minutes). 2R, 3R, and 4R rotate under bz2, but stop before or at the boundary between bz2 and the left seam hypodermal cell (Fig 8A and 8C; Table 3).

**Table 3:** R cell leading edge positions (361–433 minutes).

genotype		right side		dorsal midline	left side		
		dorsal hyp bz2	muscle	dorsal hyp bz1	muscle	dorsal hyp bz2	n
WT	int2	0	0	0	82	18	17
	int3	0	0	0	78	22	27
	int4	0	0	0	70	30	27
*unc-6(ev400)*	int2	0	40	20	40	0	15
	int3	0	30	40	30	0	20
	int4	0	11	83	6	0	18
*unc-40(e1430)*	int2	0	35	29	35	0	17
	int3	0	10	52	38	0	21
	int4	0	14	68	18	0	22
*madd2(zu475)*	int2	45	55	0	0	0	11
	int3	0	0	62	38	0	21
	int4	0	0	62	38	0	21
*unc-5(e53)*	int2	0	0	0	46	52	23
	int3	0	0	0	68	32	31
	int4	0	0	0	68	32	31

In the early E16 stage, 2L and 2R meet symmetrically at the ventral midline (Fig 8A at 249 minutes). At around 289 minutes, however, 2L invariably shifts clockwise to replace, or displace, 2R at the midline; 2L shifts for variable distances over ventral neurons (Fig 8D) and behind the valve midline cell, v3V (Fig 8E). We call this event the pre-rotation shift of 2L, as it can occur 30 or more minutes before any other evidence of rotation in the int2-4 rings. This asymmetric shift requires the LIN-12/Notch signaling pathway, as 2L and 2R remain symmetrically at or near the ventral midline in *lin-12(n941)* mutant embryos (10/10 embryos; Fig 8F). At the stage when the wild-type 2L shifts over ventral neurons, the int3 cells are separated from ventral neurons by the int2 cells (Fig 8A at 289 minutes), and the int4 cells are separated from ventral neurons by the primordial germ cells (Fig 7A at 337 minutes). The int3 and int4 cells begin to rotate clockwise soon after they first contact the ventral neurons. The int3 cells make contact when they spread ventrally, behind the int2 cells, and the int4 cells make contact when the primordial germ cells are partially engulfed by the int5 cells and/or displaced posteriorly by elongation of the intestine (Fig 7A). The rotating 2L, 3L, and 4L cells continue clockwise rotation over ventral neurons and the left ventral muscle quadrant, stopping under the right ventral hypodermal cell or left seam hypodermal cell (Figs 8A and S3D).

### Non-intestinal cells contribute to int ring rotation

**Fig 9:**
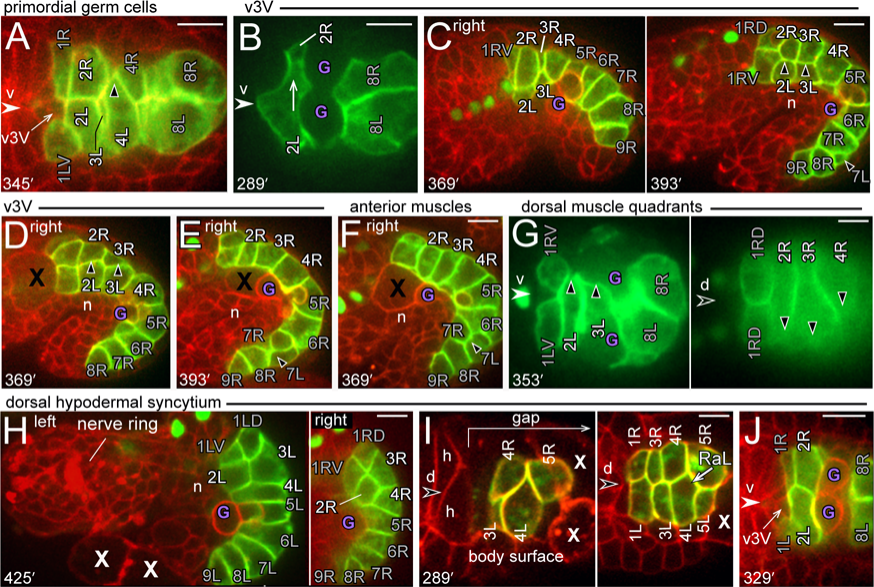
Non-intestinal cells contribute to int ring rotation. (A) Embryo following ablation of a primordial germ cell precursor (the P4 blastomere). The image shows a horizontal plane just above the ventral neurons and through the ventral surface of the primordium. 4L has premature contact with ventral neurons and rotates prematurely past the ventral midline (v). 2L has not yet rotated beyond v3V, and 3L has just reached the ventral surface. (B-E) Embryos following ablation of a v3V precursor (the MSaa blastomere). The ablated blastomere was either not internalized within the embryo (panel C), internalized but located anterior of the primordium (X in panel D), or internalized and positioned between the primordium and ventral neurons (X in panel E). Note that the int2 and int3 rings fail to rotate when they are separated from the ventral neurons (panel E). (F) Embryo following ablation of anterior muscle precursors (MSap and MSpp). The int2 and int3 rings fail to rotate, and are separated from the ventral neurons by the ablated blastomere (X) and the primordial germ cells (G). Note that the int7 ring appears to have begun the normal, counterclockwise rotation with 7L dorsal. (G) The left and right panels show the ventral and dorsal surfaces, respectively, of the primordium in a single embryo following ablation of the dorsal muscle precursors (the Cap, Cpp, and D blastomeres). The int2 and int3 rings have rotated normally, and 4R is beginning to rotate. (H-J) Embryos following ablation of the dorsal hypodermal precursors (the Caa and Cpa blastomeres). (H) Left and right sides of the primordium at 425 minutes. The int2, int3, and int4 rings have not rotated, and the int2 cells have not completed intercalation/closure. (I) The left panel shows the dorsal surface of an operated embryo at 289 minutes, and the right panel is a slightly lower focal plan of the same embryo. The ablation created a cell-free gap over the primordium, extending posterior from the int1 ring. Note that cells in the primordium have normal RaL contacts, and that there are no muscles flanking the primordium. (J) Ventral surface of an operated embryo at 329 minutes, showing the normal pre-rotation shift of 2L over ventral neurons and behind v3V. Panels = A,J [JJ2360]. Bars = 10 microns.

We used a laser microbeam to ablate various blastomeres that are precursors of cells surrounding the primordium; the precursors were identified according to the embryonic lineage data [8]. We first ablated the precursor of the primordial germ cells, which initially separates the int4 cells from ventral neurons. In each of the operated embryos, 4L contacted ventral neurons prematurely and rotated prematurely; either at the same time as, or before, 2R and 3R (2/6 cases before 2R, 4/6 before 3R; Fig 9A). These results suggest that the primordial germ cells inhibit or delay int4 rotation. To examine whether v3V was required for the pre-rotation shift of 2L, we ablated a precursor of v3V. We found that 2L usually shifted over the ventral neurons (5/6 embryos; Fig 9B), although in one embryo both 2L and 2R remained at the ventral midline. Thus, contact with v3V is not essential for the pre-rotation shift of 2L. At later stages, the operated embryos showed an interesting correlation between int ring rotation and the position of the ablated blastomere. If the ablated blastomere was outside the body or was anterior of the primordium (4/6 embryos), the int2 and int3 rings contacted ventral neurons and rotated, as in normal embryos (Fig 9C and 9D, respectively). However, if the ablated blastomere was lodged between the int rings and the ventral neurons, the int rings did not rotate (2/6 embryos; Fig 9E). We found that we could create a similar separation between the int rings and the ventral neurons by ablating precursors of anterior muscles that normally have no contact with the primordium; again, the int rings did not rotate (6/6 embryos, Fig 9F). These results support a hypothesis that int ring rotation involves contact with ventral neurons.

To examine possible roles for dorsal cells, we ablated three precursors that together form the dorsal muscle quadrants flanking the int2-4 rings (Fig 9G). Most of these embryos showed complete, or nearly complete, rotation of the int rings (5/6 embryos; the remaining embryo had severe body defects and could not be evaluated). Finally, we ablated precursors of the dorsal hypodermal syncytium that contacts the intestine primordium; these operated embryos showed little or no int ring rotation (7/7 embryos; Fig 9H). In addition, most of these embryos had a defect in int2 intercalation/closure, such that 2L and/or 2R failed to reach the dorsal midline (6/7 embryos; Fig 9H). We repeated these ablation experiments to examine earlier stages of the primordium. As predicted from the embryonic lineage, the ablations created an early, cell-free gap over most of the dorsal surface of the primordium (Fig 9I). The early events of primordium development appeared normal: The int5 cells intercalated in all of the operated embryos, and the dorsal surface of the primordium developed normal RaL asymmetry (5/5 embryos, Fig 9I). On the ventral surface, 2L showed the normal, pre-rotation shift across ventral neurons and behind v3V (4/4 embryos, Fig 9J). However, the muscle groups failed to separate into quadrants in the operated embryos, and instead all muscles clustered ventrally (Fig 9I). Thus, the intestine primordium in these operated embryos developed without the normal contacts to either the dorsal hypodermal cells or dorsal muscles. Because our results above show that dorsal muscles are not essential for rotation, we infer that rotation requires the dorsal hypodermis.

### EFN-4/Ephrin, MAB-20/Semaphorin-2a, and SAX-3 function in int2 intercalation/closure

**Fig 10:**
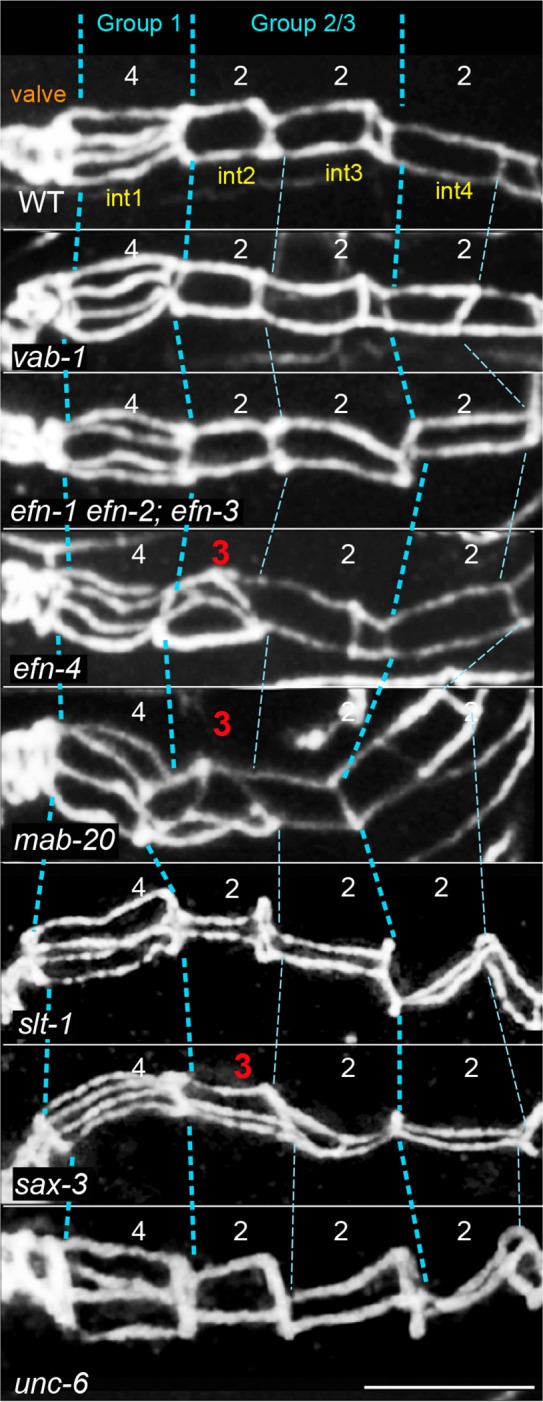
Adherens junction patterns in wild-type and mutant intestines. The images show adherens junctions (white) in the intestines of embryos with the indicated genotypes; the embryos are about 1–2 hours before hatching. The adherens junctions were stained with mAbMH27; the region shown corresponds to the int1-int5 rings in wild-type animals. In three dimensions, the adherens junction is a polygon surrounding the apical face of a cell (see Fig 1H). The contiguous adherens junctions of adjacent cells cannot be resolved by light microscopy, and so appear as a single bars of immunostaining. Thus, the 4-cell int1 ring is expected to show four longitudinal bars of staining, and each of the 2-cell int rings is expected to show two bars. The images here are maximum intensity projections to show all of the longitudinal bars simultaneously. Intestinal cells were scored in groups as explained in the legend for Table 4, and abnormal cell counts are indicated in red.

In searching for genes involved in the various aspects of intestinal morphogenesis, we examined the *C*. *elegans* Ephrin pathway. *C*. *elegan*s has a single Eph receptor (VAB-1), three conventional Ephrin (EFN-1, EFN-2, and EFN-3) and a fourth, highly diverged Ephrin (EFN-4) [29–31]. Mutants defective in any of these genes are viable, but have defects in body morphology [29–32]. We immunostained fixed embryos for adherens junctions, and scored the number of longitudinal segments as a proxy for cell number (see Legends to Fig 10 and Table 4). We found that intestine organization appeared normal in *vab-*1 mutants, in single mutants defective in any one of the ligands *efn-1*, *efn-2*, *or efn-3*, and in triple mutants defective in all three of those ligands (Fig 10, Table 4). By contrast, mutant embryos defective in the ligand *efn-4* appeared to have abnormal numbers of intestinal cells adjacent to the int1 ring (Fig 10, Table 4). Previous studies showed that the ligand EFN-4 has a role in epidermal morphogenesis that is at least partly independent of the Eph receptor VAB-1, and that appears to involve MAB-20/semaphorin-2a [32, 33]. Semaphorins are secreted and transmembrane proteins that act as short-range signals in a variety of roles, including axon guidance, cell migration, and tissue morphogenesis [34–38]. In addition to the secreted semaphorin MAB-20, *C*. *elegans* encodes two transmembrane semaphorins called SMP-1 and SMP-2 [37, 39]. We found that nearly all *mab-20* mutant embryos, but no *smp-1* or *smp-2* embryos, have a defect in intestinal adherens junctions that is very similar to that of *efn-4* embryos (Fig 10, Table 4). Plexins are a major class of transmembrane receptors for semaphorins, and *C*. *elegans* encodes two plexins, PLX-1 and PLX-2 [40, 41]. PLX-2 can bind to MAB-20 in biochemical studies, and PLX-1 appears to be expressed in intestinal cells [40, 41]. However, the pattern of intestine adherens junctions appeared normal in *plx-2* mutants, and in *plx-1 plx-2* double mutants (Table 4), suggesting that the plexins are not essential receptors for MAB-20 in intestinal morphogenesis. Axon guidance along the midline of *C*. *elegans* can involve the receptor SAX-3/Robo and the secreted ligand SLT-1/Slit [16]. We found that about half of *sax-3* mutant embryos have a defect in adherens junction patterning that appears identical to that of *efn-4* and *mab-20* mutant embryos, but that *slt-1* mutants have no apparent defects (Fig 10, Table 4). This phenotypic difference between *sax-3* and *slt-1* mutants is consistent with previous results showing that (1) *sax-3* mutants have up to 80% embryonic/larval lethality, while most homozygous *slt-1* mutant embryos are viable [16, 42], and that (2) SAX-3 can have both SLT-1-dependent and SLT-1–independent functions in axon migration [43].

**Table 4:** Percentage of cell groups with the expected, wild-type numbers^*^.

genotype	Group 1 4 cells (int1)	Group 2/3 4 cells (int2-3)	Group 4/6 6 cells (int4-6)	Group 7/9 6 cells (int7-9)	n
WT	100	98	100	98	61
*vab-1(dx31)*	100	98	91	100	54
*efn-1/vab-2(e96)*	100	100	99	100	48
*efn-2(ev658)*	100	98	98	100	42
*efn-2(ev658) efn-3(ev696)*	100	98	100	100	52
*vab-2(ju1) efn-2(ev658); efn-3(ev696)*	100	98	96	98	46
*efn-4(bx80)*	100	2	100	100	91
*mab-20(ev574)*	100	2	100	100	51
*smp-1(ev715)*	100	100	96	98	46
*smp-2(ev709)*	100	100	97	100	37
*plx-2(ev773)*	100	100	98	100	48
*plx-1(ev724) plx-2(ev773)*	100	96	93	100	45
*slt-1(eh15)*	100	100	100	100	30
*sax-3(ky123)*	100	41	98	100	61
*unc-6(ev400)*	100	100	100	100	12
*unc-40(e1430)*	100	100	100	100	21
*madd-2(zu475)*	100	94	100	100	16
*unc-5(e53)*	100	100	100	100	10

* Because abnormally patterned adherens junctions can complicate int ring identification, we binned cells into groups using the following criteria: Group 1 = intestinal cells in direct contact with the valve (normally the 4-cell int1 ring), Group 4–6 = int5 and cells in direct contact with int5 (normally int4-6; the int5 cells can be identified unambiguously as they engulf the primordial germ cells), Group 2/3 = cells between Group 1 and Group 4/6 (normally int2 and int3), Group 7/9 = all cells posterior to Group 4/6 (normally int7-9). The numbers of cell expected for the wild-type groupings are indicated in bold. Cell numbers were inferred from the number of longitudinal segments of adherens junctions, as shown in Fig 1G and 1H.

**Fig 11:**
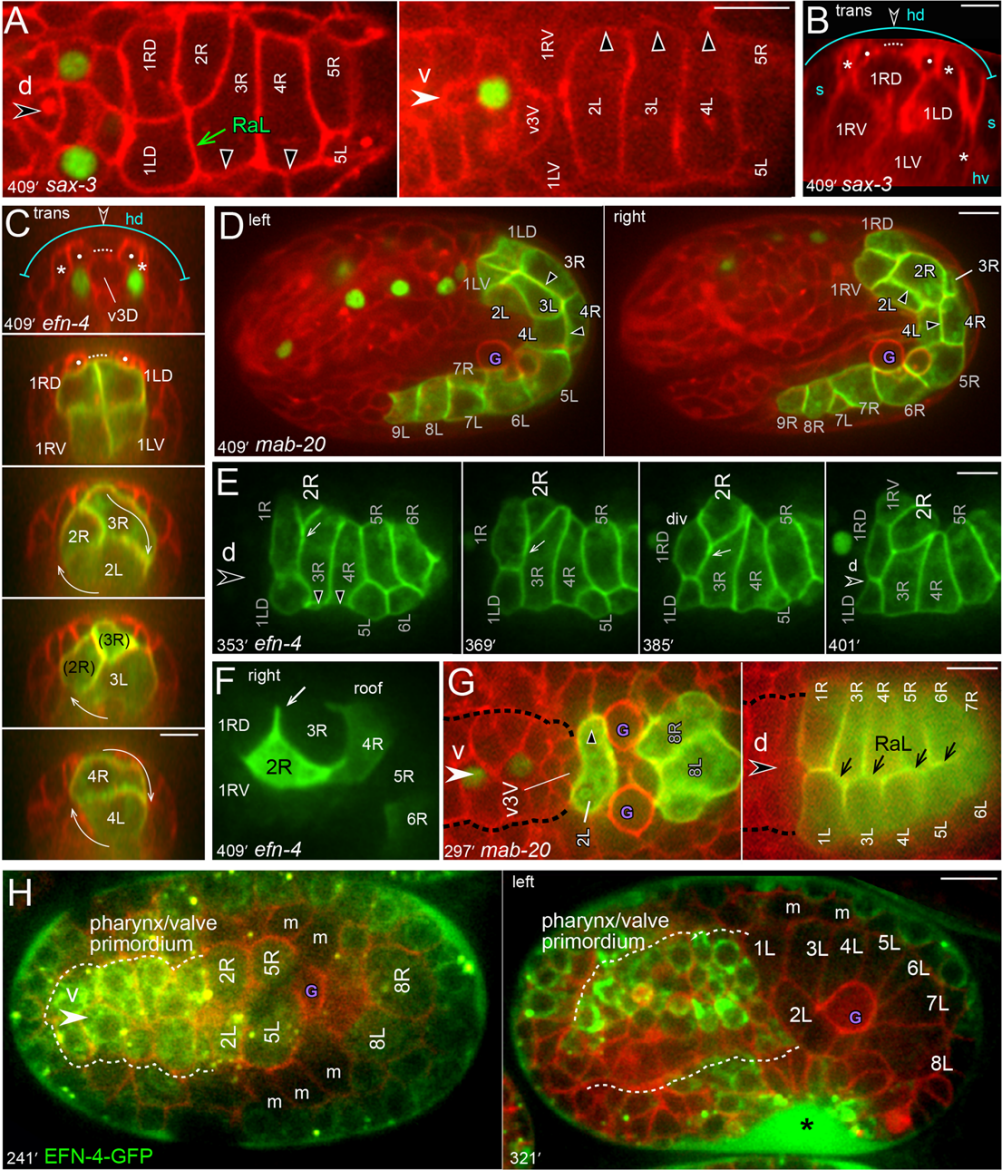
Intestinal morphogenesis defects in *efn-4*, *mab-20*, and *sax-3* mutant embryos. (A-B) Intestine primordium in *sax-3* mutant embryos; these embryos express only the general membrane reporter (red). Panel A shows dorsal and ventral sides of the E20 primordium; note that the second ring consists of three cells (2R, 3R, and 2L). The int3 and int4 rings rotated, but 2R stalled at the RaL contact between 3R and 1LD. Panel B shows a transverse view of an improperly oriented int1 ring. Both R and L cells in the int1 ring contact bz1 in most wild-type embryos (78%, n = 32) and in most *sax-3* mutant embryos (63%, n = 33). The wild-type R or L cells can cover most or all of bz1 in the remaining embryos, but never extend beyond bz1 (0/32). By contrast, R cells extended past bz1 in 14% of the *sax-3* mutant embryos. (C) Transverse projections through successive int rings in an *efn-4* mutant embryo; note that the second int ring contains three cells. (D) Left lateral view of a *mab-20* embryo showing four cells in the second int ring (3R, 3L, 2L, and 2R). (E) Dorsal view of the primordium in an *efn-4* mutant embryo. 2R intercalation stalled when 1R divided, as in wild-type embryos. However, 2R failed to resume intercalation and instead retracted. (F) *efn-4* mutant embryo expressing an *end-1*::GFP transgene from a non-integrated array. The mosaic expression in 2R shows a persistent lateral protrusion (arrow) between 1RD and 3R, but a failure in int2 intercalation/closure. (G) Early stages of the intestine primordium in a *mab-20* mutant embryo showing the normal pre-rotation shift of 2L (30/31 embryos) and normal RaL asymmetry (23/25 embryos). (H) EFN-4-GFP expression. The panel at left shows expression in the int2, int5,and int8 cells before int5 intercalation, and the panel at right shows the lack of intestinal expression at later stages. The asterisk indicates EFN-4-GFP accumulation outside the embryo, but within the eggshell. Panels = A,F [JJ2517], B,D [JJ2486], C,G [JJ2583], H [JJ2502]. Bars = 5 microns.

We crossed fluorescent reporters for intestine-specific and/or general membranes into *efn-4*, *mab-20*, and *sax-3* mutant strains to examine intestinal morphogenesis. We found that in each of the mutant embryos the abnormal pattern of adherens junctions resulted from a failure in int2 intercalation/closure. This defect resulted in a hybrid second ring with 3 cells (2L, 2R, and 3R; Fig 11A and 11C) or occasionally 4 cells (2L, 2R, 3R, and 3L; Fig 11D). In normal development, 2R intercalation breaks R to R contacts from 3R to 1RD, and breaks diagonal RaL contacts from 3R to 1LD. In the *sax-3* and *mab-20* mutant embryos, 2R usually broke the R-R contact, but did not break the RaL contact. The *efn-4* mutant embryos had a similar but more severe defect: 2R extended a lateral protrusion toward the dorsal midline, but the cell body either remained ventral, or retracted after shifting dorsally (Fig 11E and 11F).

Other aspects of intestinal morphogenesis appeared normal in the *sax3*, *mab-20*, and *efn-4* mutant embryos (see legend for Fig 11). For example, all embryos showed the pre-rotation shift of 2L over ventral neurons, and most embryos developed RaL asymmetry (Fig 11G). Both R and L cells in the int2-4 rings rotated, with the exception of 2R, which failed to cross the dorsal midline (Fig 11A). However, the centering of the int1 R-L boundary (Figs 8B and S3D) appeared defective in some *sax-3* mutant embryos (Fig 11B). The *efn-4* mutant embryos appeared to have additional, variable defects in maintaining some adhesive contacts between intestinal cells as follows. When wild-type int3 cells constrict at the beginning of int5 intercalation (Fig 4D), the *efn-4* mutant int3 cells could detach partially from each other and lose contact with the dorsal roof of the primordium (6/17 embryos; Fig 4E). Similarly, when the int4 cells constrict at the end of int5 intercalation, they could detach partially from each other (2/17 embryos). Although most *efn-4* embryos eventually developed RaL asymmetry, several appeared defective in forming and/or maintaining RaL contacts (Fig 6F).

Previous studies showed that a *mab-20*::GFP reporter is expressed in all cells of the embryo beginning at about 240 minutes [39]. EFN-4-GFP is expressed in neural or epidermal precursors beginning at about 150 minutes, but intestinal expression has not been reported [32, 40]. We crossed the latter reporter into a strain expressing a general membrane reporter, and found that EFN-4-GFP is detectable in intestinal cells prior to int5 intercalation (Fig 11H). EFN-4-GFP is not detectable in intestinal cells at later stages, but is abundant in a subset of pharyngeal and valve cells anterior to the intestine (Fig 11H). As expected for a secreted protein, EFN-4-GFP accumulates at high levels in all visible extracellular spaces within the embryo (asterisk in Fig 11H).

### Intestinal twist involves an UNC-6/netrin pathway

In an earlier report, we noticed what appeared to be only minor defects in the intestine primordium of mutants defective in axon guidance molecules such as UNC-6/netrin and its receptors UNC-40/DCC and UNC-5 [21]. As suitable fluorescent membrane reporters were not available, those early studies used DIC microscopy to infer intestinal cell rotation by nuclear positions. In the present study, we immunostained fixed *unc-6*, *unc-40*, and *unc-5* mutant embryos for adherens junctions, and found that the mutants had the wild-type number of cells in the various intestinal rings (Fig 10 and Table 4). However, we were prompted to reconsider these genes after discovering that a mutation in MADD-2 (Muscle Arm Development Defective-2) causes a penetrant defect in intestinal twist. MADD-2 is a multidomain protein with a RING finger, which functions as an E3 ubiquitin ligase; MADD-2 regulates signaling by UNC-40/DCC during *C*. *elegans* muscle development [44–46]. Briefly, we isolated a twist-defective mutant, *zu475*, from a pilot screen for intestinal morphogenesis mutants. Mapping and genome sequencing showed that the twist defect is closely linked with two candidate mutations, including an S684L missense mutation in the *madd-2* gene; the identical mutation had been described previously for the *madd-2(tr162)* allele [44]. We obtained a *madd-2(tr162)* mutant strain, and found that it has a twist defect apparently identical to that of *zu475* [hereafter, *madd-2(zu475*)].

**Fig 12:**
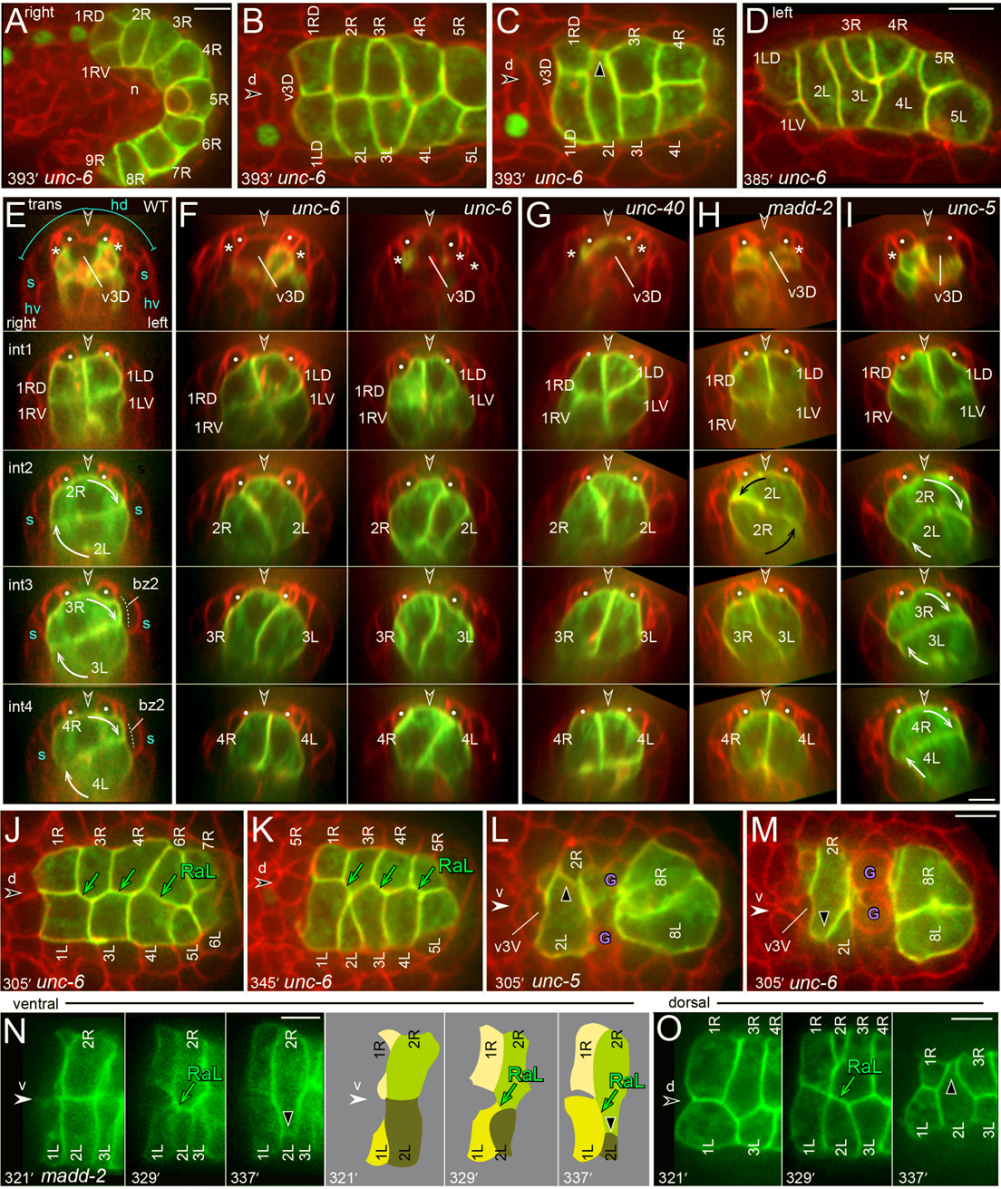
Int ring rotation defects in UNC-6/netrin pathway mutants. (A-D) Examples of defects in the E20 primordium of *unc-6* mutant embryos; similar defects are observed in *unc-40* and *madd-2* mutant embryos (Tables 3 and 5). (A) Right side of an *unc-6* primordium showing the lack of L cell rotation; note that 2L, 3L, and 4L each contacts ventral neurons (n). (B-C) Dorsal views of the *unc-6*, E20 primordium showing no rotation of the int2-4 rings (panel B) and a reversed, counterclockwise rotation of the int2 ring (panel C). (D) Left-dorsal side of the E20 primordium showing partial interdigitation of R and L cells within each of the int3 and int4 rings. (E-I) Transverse views through successive int rings in wild-type or mutant embryos, as indicated. Two *unc-6* embryos are shown to illustrate directional variation in the partial rotations of the int rings. (J, K) Dorsal surfaces of the *unc-6* primordium at the indicated times, showing normal RaL asymmetry. (L) Ventral view of an *unc-5* primordium, showing the normal, pre-rotation, clockwise shift of 2L across ventral neurons and behind v3V. (M) Ventral view of an *unc-6* primordium showing an abnormal, counterclockwise spreading of 2R over ventral neurons. (N,O) Image sequence and diagram showing the counterclockwise rotation of the int2 ring in a *madd-2* embryo; panel N shows the ventral surface of the primordium, just above ventral neurons, and panel O shows the dorsal surface of the same primordium. Note the ectopic, ventral RaL contact between 2R and 1L (panel N), and the simultaneous shift of 2R away from the dorsal midline (panel O). Panels = A-D, F, J, K, M [JJ2534], E [JJ2360], G [JJ2530], H,N,O [JJ2528], I [JJ2529]. Bars = 5 microns.

We built *unc-6*, *unc-40*, *madd-2*, and *unc-5* strains expressing fluorescent membrane reporters to examine intestinal twist. *unc-5* mutants embryos appeared to have normal intestinal twist. However, almost none of the *unc-6*, *unc-40*, or *madd-2* mutant embryos showed normal rotation of all three of the int2-4 rings (Fig 12A–12D; Table 5). We examined rotation in transverse projections of comparably staged, wild-type and mutant embryos (Fig 12E–12I). In wild-type embryogenesis, the dorsal midline valve cell, v3D, is born on the left side of the embryo, but rotates counterclockwise beneath bz1 (Fig 12E) [9]. v3D rotated normally in all *unc-6*, *unc-40*, and *madd-2* mutant embryos (n = 18–25 embryos each; Fig 12F–12H). However, v3D failed to rotate in most *unc-5* mutants, and instead remained largely on the left side of the embryo (n = 13/19 embryos; Fig 12I). The int1 ring appeared normal in all of the mutants, with both R and L cells contacting bz1. The int2-4 rings rotated normally in the *unc-5* mutants (Fig 12I; Table 5), but int ring rotation was defective in the *unc-6*, *unc-40*, and *madd-2* mutants as follows. The leading poles of 3L and 4L remained at or near the ventral midline (Fig 12F–12H). The leading poles of 3R and 4R usually remained at or near the dorsal midline (Fig 12B), but could also be shifted a short distance clockwise or counterclockwise beneath the left or right muscle quadrants (quantitated in Fig 8C and Table 3). When an L cell crossed the dorsal midline counterclockwise, the complementary R cell sometimes crossed the dorsal midline clockwise (Fig 12D). This resulted in a partial, dorsal interdigitation of R and L cells within an int ring, but did not result in mixing of cells between different int rings. Despite defects in rotation, many of the mutant rings had dorsal-ventral nuclei, similar to those of rotated, wild-type int rings (see for example the int3 ring in Fig 12F).

**Table 5:** Percentage of int rings showing full rotation between 393–441 minutes.

genotype	int2	-	int2	-	-	int2	int2	-	
	int3	-	-	int3	-	int3	-	int3	
	int4	-	-	-	int4	-	int4	int4	n
WT	83	2	1	1	0	10	0	3	96
*sax-3(ky123)*	84	1	1	0	0	9	1	3	77
*unc-5(e53)*	88	4	1	1	0	1	0	4	83
*unc-6(ev400)*	1	48	40	4	0	5	0	2	127
*unc-40(e1430)*	0	67	25	8	0	0	0	0	97
*madd-2(zu475)*	0	60	38	2	0	0	0	0	40
*madd-2(tr162)*	0	65	33	1	0	1	0	0	41

By contrast with the penetrant defects in int3 and int4 rotation, the int2 ring was fully rotated in 30–40% of the *unc-6*, *unc-40*, and *madd-2* mutant embryos (Fig 12C; Table 5). Surprisingly, we found that the direction of int2 rotation was usually reversed; counterclockwise instead of clockwise. We found that most aspects of morphogenesis appeared normal in *unc-5*, *unc-6*, and *madd-2* mutant embryos; for example, the embryos developed RaL asymmetry, int5 intercalated after int3 constriction, and the int2 cells intercalated (n = 14–26 embryos each; Fig 12J and12K). The *unc-5* mutants had the normal clockwise, pre-rotation shift of 2L over ventral neurons (Fig 12L). However, this shift did not occur in *unc-6* or *madd-2* mutant embryos, or the direction was reversed with 2R spreading counterclockwise over the ventral neurons (Fig 12M). As the int1 cells spread ventrally, prior to division (see Fig 5C), 2R made an ectopic RaL contact with 1L and spread counterclockwise (Fig 12N). This ectopic RaL contact appeared to shift the 2R cell body ventrally, such that the intercalating 2L cell reached the dorsal midline first (Fig 12O at 329 minutes), and thereafter rotated across the midline (Fig 12O at 337 minutes). These observations suggest that novel RaL contacts might contribute to the reversed, clockwise rotation of the int2 ring.

**Fig 13:**
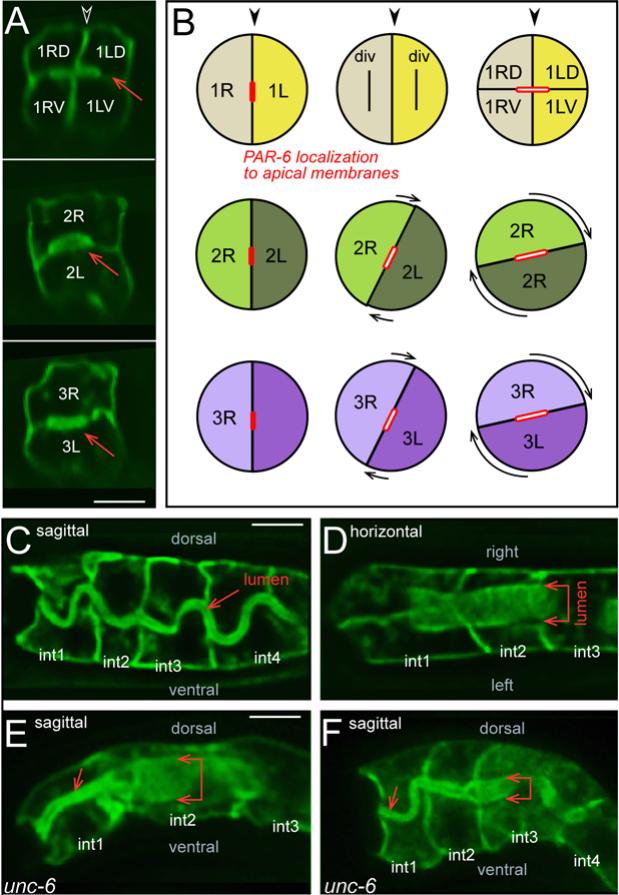
Lumen formation and int ring rotation. (A) Transverse view through successive int rings in a wild-type intestine shortly before hatching. Note that the broad axis of the closed lumen (arrow) is horizontal in the non-rotated, 4-cell int1 ring, similar to the lumen in the rotated, 2-cell int2 and int3 rings. (B) Summary diagram of lumen formation; see S4 Fig for supporting data. (C,D) Wild-type embryos showing uniform, oriented flexing of the intestinal lumen as the embryo moves in the eggshell. (E,F) *unc-6* mutant embryos showing kinks in the intestinal lumen. Note the normal alignment of the int1 lumen. Panels = A,C,D [JJ2360], E,F [JJ2534]. Bars = 5 microns.

We noticed morphological defects in the intestinal lumen of *unc-6*, *unc-40*, and *madd-2* mutant embryos near hatching. The wild-type lumen appears to develop piecemeal in the individual int rings [6, 47], and as the lumen assembles and increases in width it closes to resemble a flattened or collapsed tube (Figs 13A and S4). In late embryogenesis, the closed lumen in the int2 and int3 rings is oriented horizontally, parallel to the boundary between the R and L cells (Fig 13A). Surprisingly, we found that the closed lumen in the 4-cell int1 ring is also oriented horizontally, which is perpendicular to the boundary between the R and L cells (Figs 13A and S4A). Before the dorsal-ventral division of the 2-cell int1 ring, the nascent apical membranes of 1R and 1L are oriented vertically, similar to other 2-cell int rings (S4E Fig). After division, however, the apical membranes of the int1 daughter cells align in a horizontal plane (S4E Fig). Thus, rotation of the int2-int4 rings appears to align their respective segments of the developing lumen with that of the shifted int1 lumen (Fig 13B); when the embryo moves in the eggshell, the lumen bends smoothly in the sagittal plane (Fig 13C and 13D). The int1 lumen has the normal, horizontal orientation in *unc-6*, *unc-40*, and *madd-2* mutant embryos, but is kinked either at the int1-int2 junction (Fig 13E), or at the int2-int3 junction (Fig 13F), depending on whether the int2 ring rotated. We conclude that at least one function of int ring rotation is to align the closed lumen between the anterior int rings, and that this alignment is defective in the mutant embryos.

### MADD-2::GFP is expressed asymmetrically in L cells

**Fig 14:**
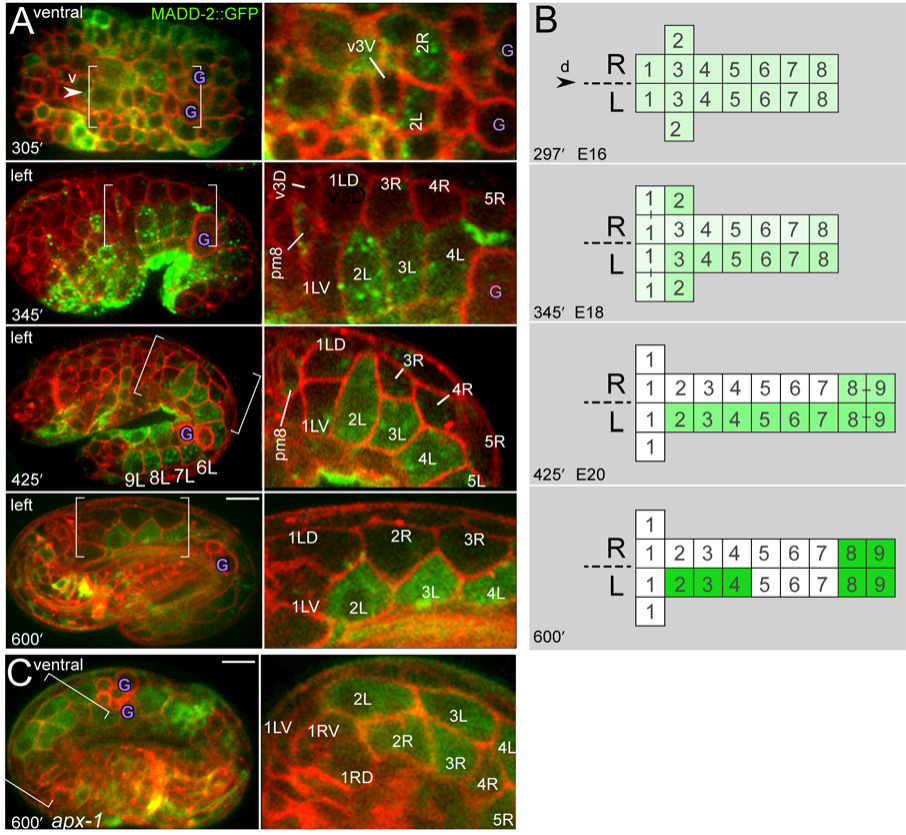
MADD-2 expression in the primordium. (A) Wild-type embryos expressing MADD-2::GFP (green) and the general membrane reporter (red) (n = 32 embryos examined). The panels at right are high magnifications of the bracketed regions. (B) Diagram summarizing changes in MADD-2::GFP levels over time, ranging from high (dark green) to low (white). Note that MADD-2::GFP disappears in both L and R cells in the int1 ring after about 345 minutes, and remains detectable in both R and L cells in the int8 and int9 rings until at least 600 minutes. MADD-2::GFP expression was not detected in the dorsal midline valve cell, v3D, which rotates counterclockwise (345 minutes), but was detectable in non-intestinal cells that move dorsally to flank v3D (pm8 at 345 minutes, see [9]). (C) MADD-2::GFP expression in an *apx-1(zu347ts)* mutant embryo following a shift to restrictive temperature (26°C) at the E8 stage. MADD-2::GFP expression disappeared in the int1 ring, as in wild-type embryos, but persisted in both R and L cells in the int2-4 rings. The image is of the ventral side of the intestine; the int2-int4 rings did not rotate, so both R and L cells are visible at the ventral midline. Panels = A,B [JJ2536], C [JJ2537]. Bar = 5 microns.

Previous studies showed that *unc-6* is expressed in the right and left ventral hypodermal cells from about 280 to 340 minutes, before these cells extend to the ventral midline (Fig 8A at 321 minutes) [48]. However, the localization of secreted UNC-6 has not been determined. The receptor UNC-40 appears to be expressed in all embryonic cells until about 100 minutes, but disappears by about 400 minutes in most cells except ventral neurons [15]. Expression of MADD-2::GFP has not been reported in intestinal cells, but is observed in ventral hypodermal cells, myoblasts, and neurons [44, 45]. We crossed a MADD-2::GFP reporter into a strain expressing the general membrane reporter, and found that MADD-2::GFP is expressed in intestinal cells in a complex and dynamic pattern (Fig 14A and 14B). MADD-2::GFP is expressed at a low level in all intestinal cells before 297 minutes, but in older embryos expression is lost in the int1 ring and in most R cells. Interestingly, MADD-2::GFP is expressed asymmetrically in the L cells of the int2-int4 rings. The second LIN-12/Notch interaction in the intestine primordium is required for int ring rotation, activates expression of the repressor REF-1/bHLH in R cells, and requires the ligand APX-1/Delta [21, 22]. We crossed MADD-2::GFP and the membrane reporter into a temperature sensitive *apx-1(zu347ts)* background, then shifted the mutant embryos to restrictive temperature at the E8 stage. Each of 23 embryos lacked int ring rotation and showed symmetrical expression of MADD-2::GFP in R and L cells (Fig 14C). These results suggest that asymmetric MADD-2 expression contributes to the different behaviors of R and L cells during rotation of the wild-type int rings.

## Discussion

In essence, intestinal morphogenesis forms a plane of cells, splits the plane into two layers, realigns all cells along a common axis, and then rotates both ends of the tube. The consecutive, orthogonal divisions of the E blastomere and its descendants contribute to the initial, planar shape of the primordium. However, skewed divisions occur that result in variant E4 and E8 cells in about 30% of the embryos. We speculate that skewed divisions might be even more frequent in embryos exposed to compressive forces. For example, several environmental stresses such as starvation or osmotic stress inhibit egg laying, such that large numbers of embryos become packed in the uterus [49, 50]. Our results suggest that VANG-1/Van Gogh, a component of the PCP pathway, functions to align any variant cells into the plane. The PCP pathway is best known for its role in creating an axis of polarity in epithelia that is orthogonal to the apicobasal axis, but PCP pathways also regulate cell intercalation during convergent extension in vertebrates, and coordinate movements of migrating cells through their environment [51, 52]. We do not know whether the presumptive interactions that align E descendants are between the E descendants themselves, or involve surrounding tissues. For example, the E8 and earlier cells extend numerous, dynamic protrusions between surrounding embryonic cells (S1 Video).

The first LIN-12/Notch interaction in the primordium occurs at the E4-E8 stages. Because all of the E4 and early E8 cells express LIN-12, one likely function of the planar array is to ensure that all of the L cells, but no R cells, contact ligand-expressing cells located left of the primordium [21, 22]. Our results suggest that this interaction initiates the asymmetric, RaL packing of cells. The 3D reconstruction of the intestinal tube shows that the basal surfaces of most intestinal cells are hexagonal; hexagonal packing can occur passively within a plane of soap bubbles, but hexagonal packing in epithelial sheets typically involves specific cell adhesion and contractility [53, 54]. Although R-L contacts within int rings appear relatively stable, as do R-R and L-L contacts between int rings, the diagonal R-L contacts between int rings initially are dynamic, and transition between LaR, Cross, and RaL patterns. We found that the early E descendants have predominately LaR diagonal contacts. This bias might occur because all left-right divisions in the early embryo place left daughters slightly anterior of right daughters [8], thus favoring LaR contacts. After the E8 stage, however, there is a global transition to RaL contacts in the intestinal primordium. The percentage of RaL contacts appears to increase as localized constrictions break LaR contacts, suggesting that RaL contacts are inherently stronger or preferentially stabilized. The primordium in *lin-12* mutant embryos contains abnormally large numbers of LaR contacts, possibly because RaL contacts are not stabilized. Thus, LIN-12/Notch signaling in L cells could regulate factors that promote RaL-specific adhesion. Our RaL nomenclature is used only for convenience, and other nomenclatures that integrate R-L with A-P differences might better comport with molecular mechanism. In hexagons with RaL packing, for example, three faces of an L cell contact the posterior int ring, while only one face contacts the anterior int ring (Fig 1J). Thus, the packing pattern could be described as the preferential adhesion of L cells for posterior int rings, and of R cells for anterior int rings. The RaL, LaR, and Cross contacts between groups of intestinal cells are reminiscent of dynamic contacts between groups of four *Drosophila* epithelial cells during convergent extension, called type 1, 2, and 3 contacts [55]. However, transitions in the *Drosophila* contacts occur by apical constriction and adherens junction remodeling, while the intestinal contacts do not appear to involve the apical surface, and occur when apical junctions are only beginning to form.

### Anterior-posterior patterning of intestinal cells

The planar E8 primordium splits to become a two-layered E16 primordium. Because the E8 cells divide anterior-posterior, this split compacts the E16 primordium. In addition, the split expands the anterior face of the primordium such that it contacts the entire posterior surface of the pharynx/valve primordium; this contact appears critical for the proper polarization of the pharynx/valve cells [9, 56]. We found that the split begins when two E8 cells shift ventrally; the daughters of those cells later intercalate to realign with the other intestinal cells. Our 3D analysis of the developing primordium shows that the split and subsequent cell intercalations each involve specific groups of anterior-posterior cells, and that cells behave differently within each group. For example, the split in the E8 primordium always begins by the dorsal constriction of the second, transverse row of cells. The int5 cells are always the first to intercalate at the E16 stage, and they follow an invariant path between the int4 and int6 cells. We described invariant changes in the shapes of the int3 and int4 cells during int5 intercalation, and propose that these changes facilitate intercalation by shifting mass away from the roof. These reciprocal movements allow intercalation to occur without changing the overall shape of the E16 primordium; this constancy is in marked contrast with the intercalating hypodermis, which expands like a sheet over underlying tissues [57].

The remarkable choreography of the intestinal cell movements suggests that unknown molecular differences subdivide the early primordium along the anterior-posterior axis. Key tasks in future studies will be to identify the anterior-posterior differences, and determine how those differences lead to cell-specific behaviors. The intestinal cells express signaling molecules such as EGL-15/FGF and CWN-2/Wnt, and EGL-15 appears to have a role in intestinal morphogenesis [25, 58]. The E blastomere, and all of its descendants, expresses the GATA transcription factors END-1 and END-3 [59, 60], but very few transcription factors have been identified that are restricted to subsets of E descendants. These include the labial-like Hox gene *ceh-13* and the Abd Hox *gene nob-1*, which are expressed in posterior E descendants [61, 62]. The transcription factor POP-1/TCF functions in combinatorial gene regulation, and is expressed asymmetrically between each pair of anterior-posterior sisters in the E8 primordium [21, 63–65]. POP-1 appears to have a role in determining the normal anterior-posterior boundaries of intestinal twist [21]; however, genes that are regulated by POP-1 in primordium have not been identified.

### Cell adhesion in the primordium

Despite the considerable repositioning of intestinal cells during morphogenesis, they do not intermingle with non-intestinal cells. Thus, the adhesive forces that maintain intestinal cell cohesion must be balanced with forces that move cells within the primordium. Our results suggest that the secreted ligands EFN-4/Ephrin and MAB-20/semaphorin-2a, and the receptor SAX-3/Robo, have roles in determining that balance. Previous studies showed that EFN-4 and MAB-20 function in the development of the *C*. *elegans* male tail, where specific subsets of neurons and structural cells group together to form structures called rays [39, 66]. Adjacent rays can fuse inappropriately in *efn-4* and *mab-20* mutant males, suggesting that EFN-4 and MAB-20 contribute to ray-specific adhesion, possibly by preventing non-specific cell contacts [33, 39, 67]. Mutations in *sax-3* do not appear to affect ray fusion, but result in defects in body morphogenesis [68]. In the intestinal primordium, mutations in *efn-4*, *mab-20*, and *sax-3* result in similar defects in int2 intercalation/closure. Although plexins can function as receptors for semaphorins in other systems [69], we found that mutants defective in the plexins PLX-1 and PLX-2 do not have intestinal phenotypes similar to *mab-20* mutants; our results are consistent with previous studies suggesting that MAB-20 interacts with a unknown, non-plexin receptor in *C*. *elegans* [40]. The phenotypic similarity of *mab-20* and *sax-3* mutants is intriguing, and it will be important in future studies to investigate possible relationships between these proteins.

Although the *efn-4*, *mab-20*, and *sax-3* mutants are defective in the intercalation/closure of the int2 ring, they show no apparent defects in the intercalation/closure of the int5 ring. In both intercalations, the trailing poles of the cells round up, suggesting that de-adhesion/contraction at the trailing pole contributes to force generation at the leading pole, and both intercalations are led by lateral protrusions, which likely generate traction forces through adhesion to flanking cell surfaces. One possible difference is that int5, but not int2, intercalation appears to be facilitated by reciprocal movements of flanking cells. A second difference is that the int2, but not int5, cells intercalate while the flanking int1 cells are either beginning, or completing, cell division. We showed that the extension of 2R and 2L stalls or stops if the adjacent int1 cell divides, and studies in other systems have shown that dividing cells can transiently reduce adhesion to their neighbors [70, 71]. Finally, int2 intercalation overlaps with elongation of the primordium, and longitudinal, pulling forces might exacerbate defects in lateral adhesion.

### Int ring rotation and axon guidance genes

Early studies on *C*. *elegans* development noted that the intestine was twisted with a reproducible handedness [20]. Any functional significance of the twist was not known, but twist has been speculated to affect the later growth of the gonad, which intertwines with the intestine [5]. We found that the intestinal lumen can be kinked in mutant embryos defect in int ring rotation, and propose that twist functions, at least in part, to align the developing lumen between consecutive int rings. The embryo develops within the fixed volume of the eggshell, and lumens in the intestine and other organs increase in width as closed or flattened structures that dilate just before or after hatching [6]. The closed lumen in the 4-cell int1 ring is oriented horizontally, possibly to link with the lumen of the 2-cell valve ring (Figs 1A and S4A). The clockwise rotation of the int2-int4 rings shifts their apical membranes toward the horizontal (S4E Fig), forming a smooth arc between the horizontal int1 lumen and the near vertical int5 lumen (Fig 1A). The int5 lumen remains vertical because the int5 cells don’t rotate, possibly to allow each cell to associate with, and partially engulf, one of the two ventral primordial germ cells [26]. The lumen orientation returns to horizontal in the posterior int rings; we showed that the int7 ring rotates counterclockwise, and presume that the int8 and int9 rings undergo similar rotations later in embryogenesis. Because worm musculature dictates dorsal-ventral bending of the body, a predominately horizontal orientation might allow the lumen to flex like a diving board in response to body contraction (Fig 13C), rather than a board set on edge.

Int ring rotation is a remarkable example of coordinated cell movements, where each cell smoothly replaces, or displaces, the complementary cell. We showed that ventral neurons and dorsal hypodermal cells appear to contribute to intestinal morphogenesis, as removing these cells or blocking their contact with the intestine prevents or delays int ring rotation. Importantly, we did not observe ventral-specific, or dorsal-specific defects in any of our ablation experiments, consistent with the view that the movements or R and L cells are strictly coupled. UNC-6/netrin is expressed in left and right ventral hypodermal cells during rotation, but the localization of secreted UNC-6 is not known. Thus, ventral neurons might have a role in presenting or concentrating secreted UNC-6, or contribute additional factors involved in rotation. Similar possibilities exist for the role of the dorsal hypodermal cells in int ring rotation.

The UNC-6/netrin signaling pathway is best known for its role in axon guidance. Several neurons extend axons circumferentially during embryonic and postembryonic development in *C*. *elegans*. Ultrastructural reconstructions of the developing nervous system show that the circumferential growth of axons begins at about 480–500 minutes [72], which is more than one hour after the int2-4 rings have completed rotation. The embryonic neurons have very small growth cones that are less than a micron in thickness and only a couple of microns in width [72], sizes comparable to the basal protrusions from rotating intestinal cells. The growth cones travel over a basal lamina that is clearly visible by both electron microscopy and immunocytochemistry. By contrast, basal lamina components such as laminin and type IV collagen show little or no accumulation near the rotating int rings [56, 73].

A rotating L cell develops a leading pole at the ventral midline, and an R cell develops a leading pole at the dorsal midline; this dorsal-ventral asymmetry between R and L cells provides a likely explanation for why rotation is clockwise. The asymmetry might result from an intrinsic chirality, as proposed for epithelial cells in the Drosophila hindgut [74], or the leading poles could be induced by local, environmental cues; most L cells appear to lack a ventral leading pole in *unc-6* mutants, suggesting that UNC-6/netrin might normally induce this pole. For example, UNC-6 can orient clustering of the receptor UNC-40/DCC, and UNC-40 can recruit F-actin effectors during anchor cell invasion in *C*. *elegans* [75]. Although UNC-40 appears to be expressed in all embryonic cells [15], we showed that MADD-2::GFP is enriched in L cells; MADD-2 binds directly to UNC-40, and appears to regulate UNC-40 activity [44, 45]. Thus, MADD-2 activity in L cells might induce a ventral leading pole in response to UNC-6. The late rotations of the wild-type, posterior int rings were not analyzed in the present study. However, at least the int7 ring undergoes a counterclockwise rotation, where the leading pole of 7L is dorsal, rather than ventral. MADD-2::GFP has a complex expression pattern in the posterior int rings, and it remains to be determined how or if the UNC-6/netrin pathway functions in the posterior cells.

The apparent coupling between R and L cell movements in wild-type int rings raises the possibility that L cell-specific defects in *unc-6* mutants could disrupt R cells indirectly. Some observations suggest that the 2L-4L cells develop an inappropriate dorsal leading pole in *unc-6*, *unc-40*, and *madd-2* mutant embryos. First, L cells can partially rotate counterclockwise past the dorsal midline, with a dorsal morphology that is the mirror image of normal R cell morphology. Second, the dorsal tips of L cells can interdigitate with the tips of R cells, as though both tips attempt to lead. One interesting possibility is that unknown dorsal cues normally induce the leading poles of R cells, and can induce dorsal leading poles in both R and L cells in the absence of UNC-6/netrin signaling. If so, this might provide an explanation for why int2, but not int3 or int4, frequently undergoes a reversed, counterclockwise rotation in *unc-6*, *unc-40*, and *madd-2* mutant embryos. In both wild-type and *unc-6* mutant embryos, RaL asymmetry ensures that 3R and 4R occupy most of the dorsal midline before rotation. However, 2R must intercalate to reach the dorsal midline. In wild-type embryos, the pre-rotation shift of 2L moves it away from the dorsal midline, and 2R usually arrives at the dorsal midline before, or at about the same time, as 2L (Fig 5B). The pre-rotation shift of 2L does not occur in the *unc-6* mutant embryos, apparently allowing 2R to make an ectopic RaL contact with 1L that shifts 2R away from the dorsal midline (Fig 12N). Thus, 2L can intercalate and occupy the dorsal midline before 2R (Fig 12O).

In conclusion, the intestine primordium provides a tractable genetic model for analyzing and modeling how cells assemble 3D structures. Important questions for future studies include how cell adhesion is established and modulated during intestinal cell movement, and the identification of factors that specify anterior-posterior patterning should provide useful tools to dissect and re-engineer cell behaviors in the primordium. Finally, the intestine is a relatively simple system for analyzing the molecular functions of known axon guidance genes, and for possibly identifying new ones.

## Materials and Methods

### Culture and strains

General nematode culture was as described [78]. All animals were grown at 20–22°C, except for *vang-1* mutants, which were grown at 25°C. Except for the JJ strains or noted otherwise, strains and alleles were obtained from the *Caenorhabditis* Genetics Center (http://cbs.umn.edu/cgc/acknowledging-cgc). The “wild-type” strain used for imaging was JJ2360, which is N2 Bristol containing reporters for intestine-specific membranes, general membranes, and for pharyngeal marginal cell nuclei. The general membrane reporter, *ItIs44*, encodes mCherry linked to a pleckstrin homology domain, which binds to phosphoinositide lipids at the plasma membrane [79]. The intestine-specific membrane reporter, *zuIs70*, encodes GFP linked to a CAAX sequence, which is targeted to the membrane by prenylation [80]. **JJ2360** was built from the strains OD70 (*ltIs44* [*pie-1*::mCherry::PH(PLC1-1) [81], SM202 (*pax-1*::HIS-GFP; *rol-6*) [82], and JJ1609 (*zuIs70* (*end-1*::GFP::CAAX; *him-8(e1489*) [80]). Unless noted otherwise below, constructions using JJ2360 transferred all three of the fluorescent reporters. Additional strains:

**JJ2323** [TH110 *(pie-1*::mCherry::PAR-6) [83] (gift from Tony Hyman) and *xnIs96* [*hmr-1*::HMR-1::GFP] [84] (gift from Jeremy Nance)], **JJ248**2 [*efn-2(ev658)* and JJ2369], **JJ2483** [*vab-2(e96)* and JJ2360], **JJ2484** [*vab-1(dx31*) and JJ2360], **JJ2486** [*efn-4(bx80)* and JJ2360], **JJ2492** [*lin-12(n941)/unc-32(e189)* and JJ2360], **JJ2502** [EFN-4-GFP built from CZ1566 (*lin-15B(n765) juIs109*) and OD70],

**JJ2515** [*vang-1(tm1422)* and JJ2360), *nnIs*[*unc-119(+) pie-1 promoter*::*gfp*::*Dm-moesin*^*437-578*^[76] gift from Fabio Piano], **JJ2517**[*mab-20(ev574)* and JJ2360], **JJ2521** [*plx-1(ev724);plx-2(ev773)* and JJ2360], **JJ2528** [*madd-2(zu475)* and JJ2360], **JJ2529** [*unc-5(e53)* and JJ2360)], **JJ2530** [*unc-40(e1430)* and JJ2360], **JJ2531**[*tol-1(nr2013)* and JJ2360)], **JJ2532** [*sax-3 (ky123)* and OD70, SM202], **JJ2534** [*unc-6(ev400*) and JJ2360],], **JJ2536** [RP835(MADD-2-GFP) gift from Peter Roy and OD70], and **JJ2537** [*apx-1(zu347ts)*, RP835(MADD-2-GFP), and OD70]. Other mutant alleles examined are as follows: *apx-1(zu347ts)V*, *efn-1/vab-2(e96)IV*, *efn-2(ev658)IV*, *efn-2(ev658)IV; efn-3(ev696)X*, *efn-4(bx80)IV; him-5(e1490)V*, *lin-12(n941)*, *mab-20(ev574)*, *madd-2(tr162)*V; *tris25*;*rrf-3(pk1426)*II (gift from Peter Roy), *madd-2(zu475)V*, *mab-20(ev574) (*gift from Joe Culotti), *smp-1(ev715)I; jcIs1 IV; him-5(e1490)V*, *smp-2(ev709)I; jcIs1 IV; him-5(e1490)V*, *plx-2(ev773); him-5(e1490)V*, *plx-1(ev724)IV; plx-2(ev773)II*, *plx-1(nc37) IV; him-5(e1490)V*, *plx-2(ev773) II; him-5(e1490) V*, *sax-3(ky123)X*, *smp-1(ev715) I; jcIs1 IV; him-5(e1490) V*, *smp-2(ev709) I; jcIs1 IV; him-5(e1490) V*, *slt-1(eh15)X*, *unc-5(e53)IV*, *unc-6(ev400)X*, *unc-40(e1430)I*, *vab-1(dx31)II*, *vab-2(ju1) efn-2(ev658)IV; efn-3(ev696)X*, *tol-1(nr2013)I*.

### Imaging

Embryos selected for imaging were dissected from adult hermaphrodites and allowed to settle in dH20 onto a microscope dish (Delta T dish, Bioptechs) that was coated with polylysine (Sigma). This technique avoids compression and the severe distortion of the embryo associated with conventional mounting methods between an agar pad and a coverslip [8]. Time-lapse movies were acquired with a Hamamatsu C9100-13 camera on a Nikon TE-2000 inverted microscope equipped with a Yokogawa CSU-10 spinning disk and operated with Volocity 5.3.3 software (Improvision). For analysis, the first timepoint in an image sequence was matched to the closest timepoint in the reconstructed primordium (S1 Video). Developmental events used for staging included the contraction of the int3 cells, intercalation of int5 and int2, and the division of the int1 and int8 cells. Additional staging involved events described in S3D Fig. For the analysis of tissues surrounding the intestine (Figs 8A and S3D), we used orthogonal projections of optical Z-stacks generated with ImageJ software [85, 86]. Orthogonal, high-resolution projections with the mCherry reporter used for general membranes required a much higher laser intensity that for typical timelapse imaging. Hence, we generated a reference library of single timepoint, optical stacks through 104 embryos from about 249–377 minutes. These embryos were used to assess developmental variability over the time interval, and allowed us to use non-intestinal tissues to confirm developmental times in low resolution, timelapse imaging; for example, the intercalation, spreading, and fusion of hypodermal cells.

### Reconstruction of the primordium

The confocal optical stacks used for the U13 reference reconstruction shown in S1 Video were taken with a 60X water immersion objective (Nikon), and consisted of 49 Z-slices at a step size of 0.5 microns. Additional U11 and U12 reconstruction were made from other embryos imaged for shorter time intervals with a 40X water immersion objective (Nikon); these reconstructions appeared identical to the U13 reconstruction. The events shown in the reconstruction are also supported by 44 recordings of live embryos covering all or part of the reconstructed interval (S3 Video and S4 Video); the main variation is in int2 intercalation/closure, as described in the text. Cell contours were traced using a Wacom bamboo pad and the TrakEM2 program in Fiji software [87]. The reconstruction of the intestine in a newly hatched larva used an optical stack taken with a 40X water immersion objective (Nikon), and consisted of 49 Z-slices at 0.5 microns. Object files were colored and analyzed using Blender (version 2.6) open source 3D graphics and animation software (https://www.blender.org/). We developed a “shadow” technique to estimate cell contact areas within the reconstructed primordium, based on the sophisticated light path tracking available with the Cycles Render option in Blender 2.6 (S2 Fig). Our basic approach is to surround two objects of interest with light from all directions, at an intensity such that only direct contact between the objects can prevent light from reaching a given surface. Blender software was used to draw an empty 3D rectangular box that was slightly larger than the model of the primordium; this box was designated to function as a light-emitting source. The Blender camera was placed into the light box, along with two calibration cubes, A and B, which were each about the size of an E16 cell. All light rays from cube B to the camera were selected for exclusion in the rendered image. Thus, the rendered image includes only cube A and any shadow cast by cube B. The A and B cubes were then separated by 0.5 microns, the thickness of our optical sections, and the intensity of the light box was increased until B was unable to cast a shadow on A. Finally, the calibration cubes were removed, and the 3D primordium was placed into the light box. All cells in the primordium were selected to be invisible to the camera, other than a designated pair of R and L cells. The visibility and rendering attributes of the R cell were then set as above for the calibration cube B. After rendering, images were imported into Fiji [85, 86], and the areas of shadows were measured after thresholding.

### Molecular identification of *madd-2(zu475)*

The *madd-2(zu475)* mutant was isolated in a pilot screen from the progeny of a pool of 100 F1, JJ2360 adults following standard EMS (EthylMethanesulfonate; Sigma) mutagenesis [78]. The mutagenized animals expressed both the intestine-specific and general membrane reporters described above, and mutant embryos were scored by fluorescence microscopy and recovered from microscope slides. The mutant was mapped to LGV by conventional genetic techniques. Published guidelines were followed for WGS and SNP mapping [88]. Briefly, 25 F2 recombinant worms were selected from a cross between the linkage-mapped mutant (Bristol background) and CB4856 Hawaiian males; these were allowed to self-fertilize, and their F3 and F4 progeny were pooled together. DNA was prepared (Truseq DNA kit), and samples sequenced using a Illumina HiSeq 2500 platform. Sequence analysis was with CloudMap’s Hawaiian Variant Mapping with the WGS Data tool as described [89]. This analysis identified two candidate genes, *let-413* and *madd-2*.

### Immunostaining of adherens junctions

Embryos shown in Fig 10 and described in Table 4 were fixed and stained as follows. Gravid adults were dissected on microscope slides in a small drop of M9 buffer and covered with a coverslip. The slide was frozen on dry ice, the coverslips removed, and the slide immersed in −20°C MeOH for 5 minutes, then rinsed in three changes of PBS for 5 minutes each. Slides were incubated overnight at 4°C with the antibody MH27, which recognizes the adherens junction component AJM-1 (Developmental Studies Hybridoma Bank).

### Cell ablations

A 440-nm laser microbeam (Photonics Instruments) was used for all ablations. Embryos were mounted on agar pads and covered with a coverslip [8]. Immediately following ablation, the coverslip was removed to allow the embryos to develop without compression, and the slide was placed in a humidified chamber. After the appropriate time interval, the embryos were covered with a coverslip and analyzed.

## Supporting Information

S1 Fig Variation in the two modes of int2 intercalation/closure. Each column shows sequential frames at 8-minute intervals from a movie of a single, wild-type primordium, imaged about 1 micron below the dorsal roof. Sequences in panels A-E begin when either 2R or 2L reaches the roof, and end when the int2 ring closes. (A-C) Variations in mode 1. The fastest intercalation/closure occurs when 2R and 2L reach the dorsal midline at the same time and before an adjacent int1 cell divides (Panel A). If 2L reaches the dorsal midline before 2R (panel B), it stops without crossing the midline; this contrasts with mode 2, where 2R is able to rotate across the dorsal midline. If 3R has a large RaL contact with 1L (panel C), an intercalating 2L typically stops at the RaL contact. (D-F) Variations in mode 2. If 2R reaches the dorsal midline before the adjacent 1R cell divides (panel D), 2R continues across the dorsal midline to close the int2 ring. If 2R fails to reach the dorsal midline before 1R divides (panel E), 2R temporarily stalls or retracts, but then continues across the dorsal midline to close the int2 ring. (F) Variation where 3R rotation occurs before int2 intercalation. Note that 3R has a lateral protrusion (arrow) as well as the broad, basal protrusion (triangle); the lateral protrusion retracts or collapses when 1L divides, but the basal protrusion remains in place or advances. Panels = A-E [JJ2360]. Bars = 5 microns.
(TIF)

S2 Fig Analysis of lateral surface contacts in the 3D reconstruction. (A) Oblique, surface views of the reconstructed primordium at 393 minutes. RaL contacts, but no LaR contacts, are visible between the int4 and int3 rings (left panel) and between the int6 and int5 rings (right panel). (B) Same views as panel A, but after removing the 3L and 5L cells. Note that int4, but not int6, had an additional, internal LaR contact (4L to 3R). (C) Quantitation of intestinal cell volumes over time. Previous studies showed that intestinal cell volumes increase sometime before hatching [77], which might increase the surface areas engaged in RaL or LaR contacts. However, no significant volume increase is evident within the time interval covered by the reconstruction. The starting volume of each cell is indicated in white, and fluctuations in volume are relative to the vertical scale shown. (D, E) Lateral surface areas with RaL (solid lines) or LaR (dotted lines) contacts, estimated using a 3D graphic technique described in Materials and Methods. All of the posterior int rings show a rapid conversion to RaL. The anterior int rings int3 and int4 show a similar conversion to RaL until about 313 minutes, when they begin to rotate (panel E). The int2 ring is unique in showing little or no RaL contact before rotation. After rotation, int2 makes equal RaL and LaR contacts with the 4-cell int1 ring; the latter contacts are shown in Fig 1A, where 2R contacts 1LD (RaL) and 2L contacts 1RV (LaR); see also Fig 5B.
(TIF)

S3 Fig Int ring rotation and surrounding tissues. (A-B) Supporting evidence that int ring rotation involves basal protrusions that extend transiently over complementary cells. Panels A and B are orthogonal projections of the same primordium shown in Fig 7B, but taken at a step size of 0.2 microns instead of 0.5 microns, and after deconvolution of the confocal stack; the int4 ring is rotating clockwise. In the line scan indicated by the red line, the intensity doubles where 4R contacts 4L, corresponding to the combined fluorescence from two, adjacent membranes. A similar increase in intensity is seen in line scan across the region we interpret as the leading edge of 4R (blue line). The merged panel in B shows how the shape of the leading edge changes from anterior (slice b, red) to posterior (slice a, green) in this ring (see Fig 7B). Rotation advances anterior to posterior, and other int rings have an anterior profile that resembles slice a, and a posterior profile that resembles slice b. Thus, these images suggest a model where the basal protrusion extends over the complementary cell, fills with cytoplasm, and then re-extends. (C-D) Examples of supporting data for Fig 8A, with the summary diagram redrawn for reference. The images in panel D are examples from a library of single optical stacks through 104 different embryos, ranging from 260 to 433 minutes in age. The embryos were imaged without compression (see Materials and Methods), and only a single timepoint was collected from each embryo because of the high laser intensity required for the orthogonal projections. Each column begins with a orthogonal slice through the dorsal valve cell, v3D. v3D is initially on the left side of the primordium (see Fig 8B), but by 305 minutes v3D has rotated counterclockwise and is centered under the dorsal midline (arrowhead). The lower panels for each column show successive projections through the int1-int4 rings. For example, the cells labeled R and L (grey letters) in the int2 row at 305 minutes are 2R and 2L; because int2 has not finished intercalation, 3R and 3L (labeled black) are also visible in this slice. Labels are positioned directly over the nuclei for the int2-4 rings; note that the R and L nuclei rotate from near horizontal to near vertical over time. Individual dorsal hypodermal cells fuse into a syncytium at about 289 minutes. Dividing myoblasts flank the left and right sides of the primordium at 249 minutes, and later produce the distal and proximal muscles in the muscle quadrants. In live imaging, we noticed that myoblasts next to the int1-4 intestinal cells, but not in other regions of the body, exited the mitotic cycle up to an hour before other myoblasts, and became distal muscles. A similar conclusion can be inferred by comparing published lineage charts and body diagrams [8]; these divisions are easy to score and useful reference points. The right and left distal muscles insert processes into the boundary between the dorsal hypodermal cell and the right or left seam cell (red arrow at 289 minutes in panel C). The distal muscles then elongate longitudinally, and extensions from multiple distal muscles can appear in a single transverse section through a muscle quadrant. The dorsal hypodermal cell spreads over and across the distal muscles, transferring cytoplasm laterally as the bz1 zone narrows. R cells in contact with bz1 typically become convex, bulging into the dorsal hypodermal cell; for example, note the 3R cell at 337 minutes. Conventional mounting procedures that compress the embryo greatly enhance this bulge if the embryo is on a lateral side, or eliminate the bulge if the embryos is dorsal/ventral. Compression might affect the timing of int ring rotation, but was not analyzed in this study. The rotation of an R cell appears to stop, or stall, abruptly when it reaches a hypodermal seam cell (433 minutes). Contact between the seam cells and the primordium varies depending on whether they are separated by the dorsal and ventral hypodermal cells (see Fig 1B). Panels = JJ2360. Bars = 5 microns.
(TIF)

S4 Fig Lumen development. (A-E) Supporting data for Fig 13B. (A) Electron micrograph of a transverse section through the 4-cell int1 ring in a late embryo near hatching; VNC = ventral nerve cord. Arrowheads indicate the adherens junctions flanking the apical membranes. The apical membranes have differentiated numerous microvilli, which are covered by a glycocalyx (white border). The lumen is closed, with all four apical membranes oriented to face the same, horizontal plane. (B) Orthogonal projections of the larval intestine at the indicated times after hatching, showing the int1 ring and the int2 ring. The lumen can remain closed for a short time after hatching, as shown at 20 minutes, or is already open. The lumen thereafter grows symmetrically. The larvae shown here were imaged without compression; with a conventional microscopic preparation on agar pads, the lumen appears oriented dorsal-ventral as an artifact of lateral compression. (C,D) Electron micrographs of sections through the int2 ring (panel C) and the int1 ring (panel D) at the approximate times indicated. The lumen develops from a small separation between cells, and the adherens junctions (arrows) flanking the lumen spread apart as the apical membrane increases in size and microvilli are added (see also [6]). The lumen in all int rings flattens as the lumen increases in width, creating the “closed” appearance. (E) Developmental sequence of embryos at the indicated times showing PAR-6-mCherry (red) localization to the apical membranes of intestinal cells; the embryos also express a reporter for HMR-1/E-cadherin (green) for spatial reference. The panels at right are orthogonal projections along the slices indicated at left (dashed lines). The 289 minute timepoint shows vertical PAR-6 localization in the 2-cell int1 ring, similar to that in other int rings; at this stage, the int2 cells have not intercalated, and the int3 cells lie above the int2 cells. Previous studies showed that PAR-6 disappears from the int1 membranes during division [9]; the timepoint at 361 minutes shows the int1 ring shortly after division, with horizontal PAR-6 localization. Note that the clockwise rotation of the int3 ring aligns the plane of PAR-6 with that of the int1 ring. Bars = (B-D) 5 micron, (G) 2.5 microns. Panels = B-D [JJ2360], G [JJ2323]
(TIF)

S1 Video Movie of the reconstructed primordium from the E8 to E20 stages. The top half of the frame shows the dorsal surface of the primordium and the bottom half shows the left surface; color coding as in Fig 1E. Key frames are presented with brief descriptions in Fig 2. The intestine and the body of the embryo begin elongating at about 377 minutes, and the body begins muscle movements at 409 minutes. The start of int ring rotation is visible at 313 minutes by the apparent dorsal shift in the positions of 3R and 4R (light purple and light blue, respectively). This bulge protrudes between the dorsal muscle quadrants and into the overlying dorsal hypodermal cell, and can be seen in the transverse views of embryos shown in S3D Fig between 321 and 337 minutes.
(MOV)

S2 Video Reconstruction of the intestine of a newly hatch larva. The first frame shows the left side of the intestine, dorsal up. Note that cells such as 3R and 4R are hexagons.
(MOV)

S3 Video Video showing 5R intercalation. The first frame shows the right side of the E8 primordium; 2/5R has constricted dorsally, and 1/3R has engulfed a cell death (MSpaapp). 5R intercalation begins at 273 minutes; note the dorsal constrictions of 3R (beginning at 265 minutes) and 4R (beginning at 297 minutes).
(MOV)

S4 Video Video showing 2R intercalation. The embryo is mounted without compression, so 8R is not visible in the initial frames. The reporter expressed in pharyngeal nuclei become visible at about 297 minutes, and were used for spatial and temporal reference throughout this study. The nuclei have threefold, radial symmetry relative to the lumen, with a dorsal right row (shown), a dorsal left row, and a ventral row. The focal plane selected shows the lateral protrusion (arrow at 289 minutes) that leads 2R intercalation. 2R reaches the dorsal roof of the primordium at 345 minutes, but undergoes little longitudinal expansion at the perimeter until 393 minutes, by which time the intestine and body are elongating. Similarly, 2L shows little longitudinal expansion by 417 minutes.
(MOV)

S5 Video Surface views of the reconstruction at t = 313 minutes, showing RaL contacts for all cells. The first frame shows a dorsal view of the primordium, then flips to show a lateral view, followed by rotation of the primordium to show all surface contacts.
(MOV)

S6 Video Anterior views of the reconstructed primordium (left), after removal of the int1 cells (middle), and after removal of the int2 cells (right). The movie shows that the int1 cells spread ventrally, then divide without rotating. The 2R and 2L intercalate and close together to form the int2 ring (t = 345 minutes; mode 1), then begin a clockwise rotation. The int3 cells begin rotation at 329 minutes. Note that the posterior int rings show no rotation over this period.
(MOV)
