# Correction: Morphogenesis of the *C*. *elegans* Intestine Involves Axon Guidance Genes

**DOI:** 10.1371/journal.pgen.1006077

**Published:** 2016-05-18

**Authors:** Alparslan Asan, Stephan A. Raiders, James R. Priess

The first author’s name is spelled incorrectly. The correct name is: Alparslan Asan.

## References

[1] Asan A, Raiders SA, Priess JR (2016) Morphogenesis of the *C. elegans* Intestine Involves Axon Guidance Genes. PLoS Genet 12(4): e1005950 doi:10.1371/journal.pgen.1005950 27035721 10.1371/journal.pgen.1005950 PMC4817974

