# Supplementary figures and images for: Morphogenesis of the C. elegans Intestine Involves Axon Guidance Genes
Source: PLoS Genet. 2016 Apr 1;12(4):e1005950. doi: 10.1371/journal.pgen.1005950 (PMC4817974; doi:10.1371/journal.pgen.1005950)

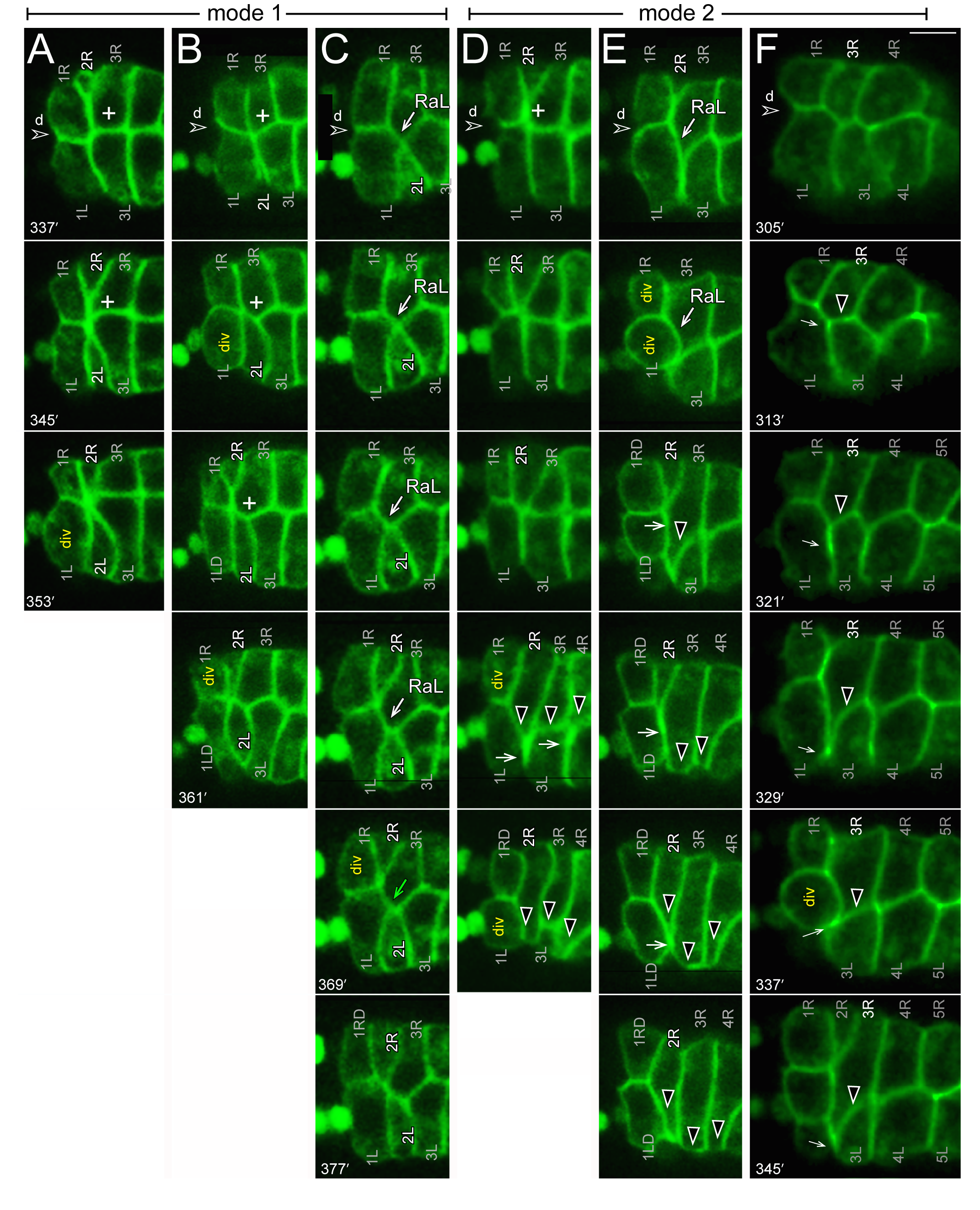

Supplement: S1 Fig — Each column shows sequential frames at 8-minute intervals from a movie of a single, wild-type primordium, imaged about 1 micron below the dorsal roof. Sequences in panels A-E begin when either 2R or 2L reaches the roof, and end when the int2 ring closes. (A-C) Variations in mode 1. The fastest intercalation/closure occurs when 2R and 2L reach the dorsal midline at the same time and before an adjacent int1 cell divides (Panel A). If 2L reaches the dorsal midline before 2R (panel B), it stops without crossing the midline; this contrasts with mode 2, where 2R is able to rotate across the dorsal midline. If 3R has a large RaL contact with 1L (panel C), an intercalating 2L typically stops at the RaL contact. (D-F) Variations in mode 2. If 2R reaches the dorsal midline before the adjacent 1R cell divides (panel D), 2R continues across the dorsal midline to close the int2 ring. If 2R fails to reach the dorsal midline before 1R divides (panel E), 2R temporarily stalls or retracts, but then continues across the dorsal midline to close the int2 ring. (F) Variation where 3R rotation occurs before int2 intercalation. Note that 3R has a lateral protrusion (arrow) as well as the broad, basal protrusion (triangle); the lateral protrusion retracts or collapses when 1L divides, but the basal protrusion remains in place or advances. Panels = A-E [JJ2360]. Bars = 5 microns. (TIF) [file pgen.1005950.s001.tif]

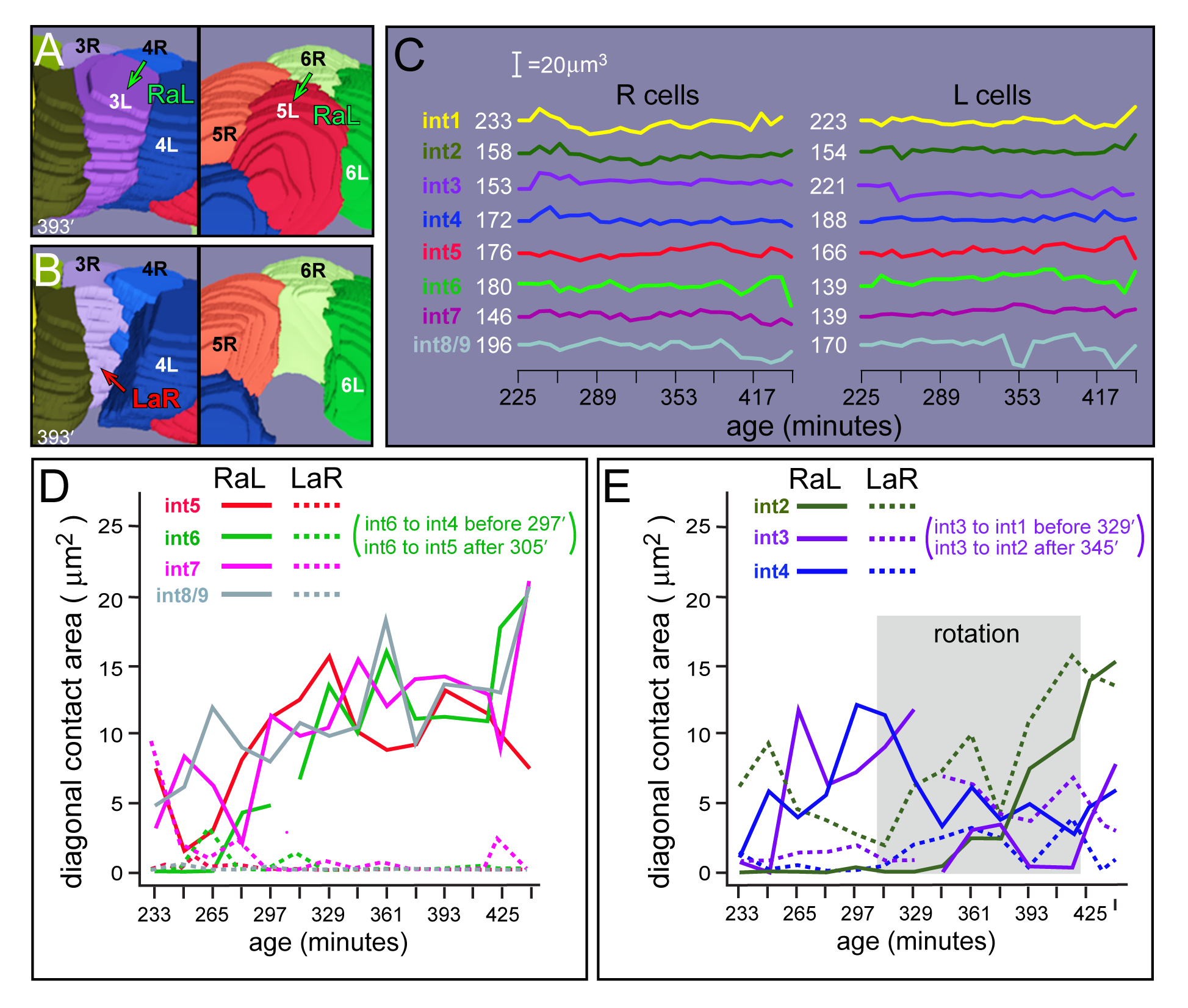

Supplement: S2 Fig — (A) Oblique, surface views of the reconstructed primordium at 393 minutes. RaL contacts, but no LaR contacts, are visible between the int4 and int3 rings (left panel) and between the int6 and int5 rings (right panel). (B) Same views as panel A, but after removing the 3L and 5L cells. Note that int4, but not int6, had an additional, internal LaR contact (4L to 3R). (C) Quantitation of intestinal cell volumes over time. Previous studies showed that intestinal cell volumes increase sometime before hatching [77], which might increase the surface areas engaged in RaL or LaR contacts. However, no significant volume increase is evident within the time interval covered by the reconstruction. The starting volume of each cell is indicated in white, and fluctuations in volume are relative to the vertical scale shown. (D, E) Lateral surface areas with RaL (solid lines) or LaR (dotted lines) contacts, estimated using a 3D graphic technique described in Materials and Methods. All of the posterior int rings show a rapid conversion to RaL. The anterior int rings int3 and int4 show a similar conversion to RaL until about 313 minutes, when they begin to rotate (panel E). The int2 ring is unique in showing little or no RaL contact before rotation. After rotation, int2 makes equal RaL and LaR contacts with the 4-cell int1 ring; the latter contacts are shown in Fig 1A, where 2R contacts 1LD (RaL) and 2L contacts 1RV (LaR); see also Fig 5B. (TIF) [file pgen.1005950.s002.tif]

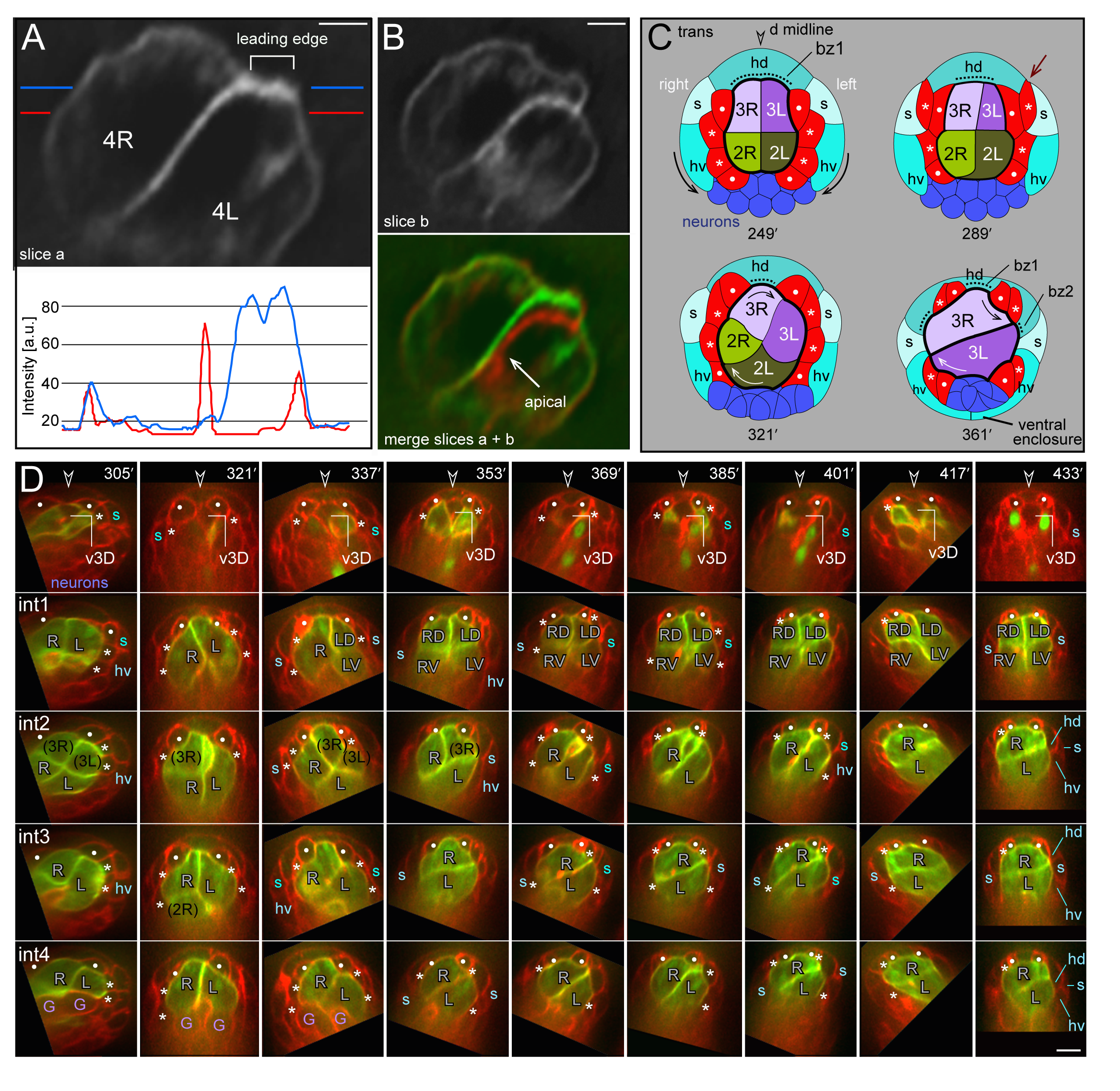

Supplement: S3 Fig — (A-B) Supporting evidence that int ring rotation involves basal protrusions that extend transiently over complementary cells. Panels A and B are orthogonal projections of the same primordium shown in Fig 7B, but taken at a step size of 0.2 microns instead of 0.5 microns, and after deconvolution of the confocal stack; the int4 ring is rotating clockwise. In the line scan indicated by the red line, the intensity doubles where 4R contacts 4L, corresponding to the combined fluorescence from two, adjacent membranes. A similar increase in intensity is seen in line scan across the region we interpret as the leading edge of 4R (blue line). The merged panel in B shows how the shape of the leading edge changes from anterior (slice b, red) to posterior (slice a, green) in this ring (see Fig 7B). Rotation advances anterior to posterior, and other int rings have an anterior profile that resembles slice a, and a posterior profile that resembles slice b. Thus, these images suggest a model where the basal protrusion extends over the complementary cell, fills with cytoplasm, and then re-extends. (C-D) Examples of supporting data for Fig 8A, with the summary diagram redrawn for reference. The images in panel D are examples from a library of single optical stacks through 104 different embryos, ranging from 260 to 433 minutes in age. The embryos were imaged without compression (see Materials and Methods), and only a single timepoint was collected from each embryo because of the high laser intensity required for the orthogonal projections. Each column begins with a orthogonal slice through the dorsal valve cell, v3D. v3D is initially on the left side of the primordium (see Fig 8B), but by 305 minutes v3D has rotated counterclockwise and is centered under the dorsal midline (arrowhead). The lower panels for each column show successive projections through the int1-int4 rings. For example, the cells labeled R and L (grey letters) in the int2 row at 305 minutes are 2R and 2L; because int2 h [file pgen.1005950.s003.tif]

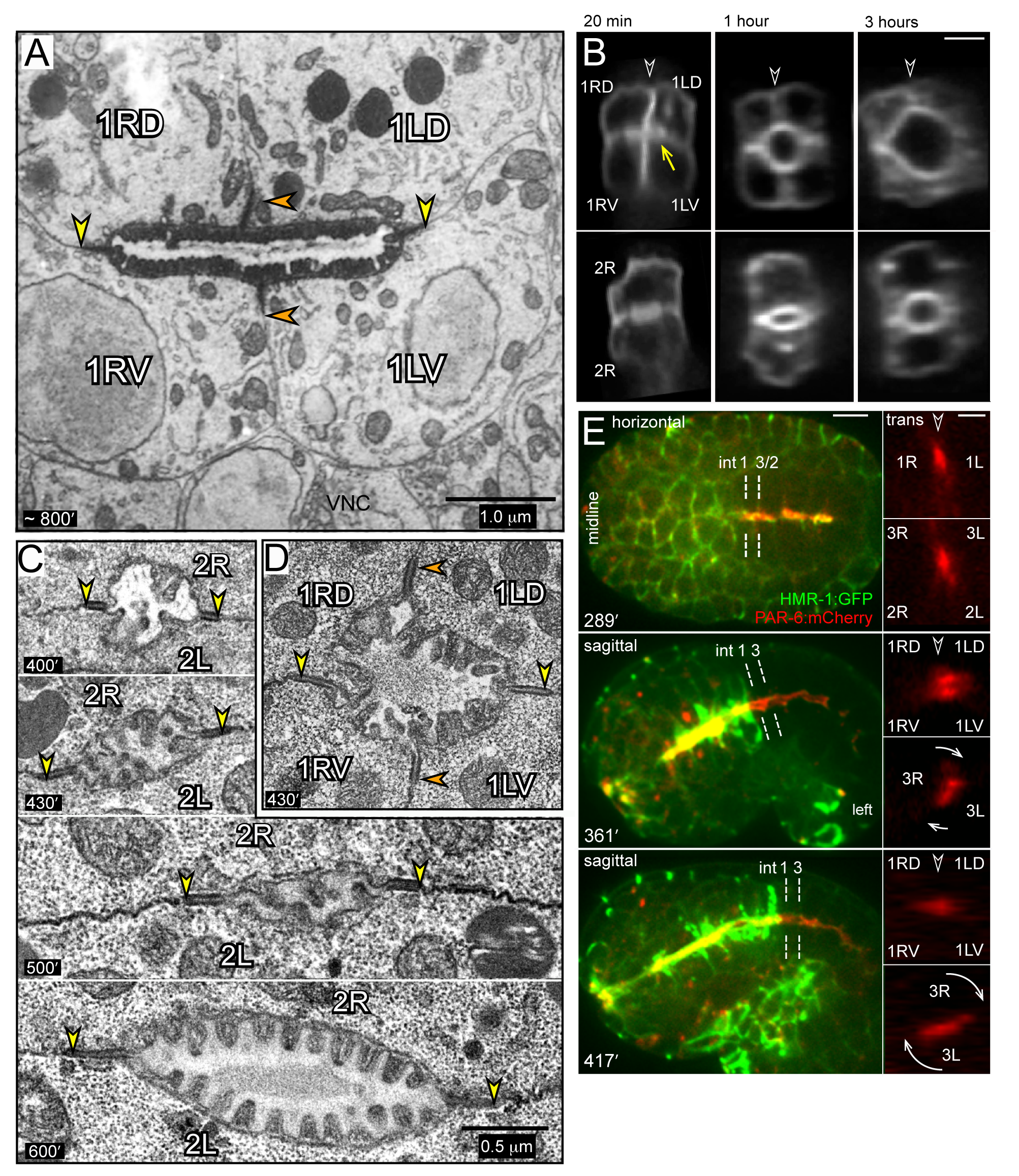

Supplement: S4 Fig — (A-E) Supporting data for Fig 13B. (A) Electron micrograph of a transverse section through the 4-cell int1 ring in a late embryo near hatching; VNC = ventral nerve cord. Arrowheads indicate the adherens junctions flanking the apical membranes. The apical membranes have differentiated numerous microvilli, which are covered by a glycocalyx (white border). The lumen is closed, with all four apical membranes oriented to face the same, horizontal plane. (B) Orthogonal projections of the larval intestine at the indicated times after hatching, showing the int1 ring and the int2 ring. The lumen can remain closed for a short time after hatching, as shown at 20 minutes, or is already open. The lumen thereafter grows symmetrically. The larvae shown here were imaged without compression; with a conventional microscopic preparation on agar pads, the lumen appears oriented dorsal-ventral as an artifact of lateral compression. (C,D) Electron micrographs of sections through the int2 ring (panel C) and the int1 ring (panel D) at the approximate times indicated. The lumen develops from a small separation between cells, and the adherens junctions (arrows) flanking the lumen spread apart as the apical membrane increases in size and microvilli are added (see also [6]). The lumen in all int rings flattens as the lumen increases in width, creating the “closed” appearance. (E) Developmental sequence of embryos at the indicated times showing PAR-6-mCherry (red) localization to the apical membranes of intestinal cells; the embryos also express a reporter for HMR-1/E-cadherin (green) for spatial reference. The panels at right are orthogonal projections along the slices indicated at left (dashed lines). The 289 minute timepoint shows vertical PAR-6 localization in the 2-cell int1 ring, similar to that in other int rings; at this stage, the int2 cells have not intercalated, and the int3 cells lie above the int2 cells. Previous studies showed that PAR-6 disappears from the int1 membranes during d [file pgen.1005950.s004.tif]
